# Stem Cell Therapies for Progressive Multiple Sclerosis

**DOI:** 10.3389/fcell.2021.696434

**Published:** 2021-07-09

**Authors:** Jayden A. Smith, Alexandra M. Nicaise, Rosana-Bristena Ionescu, Regan Hamel, Luca Peruzzotti-Jametti, Stefano Pluchino

**Affiliations:** ^1^Cambridge Innovation Technologies Consulting (CITC) Limited, Cambridge, United Kingdom; ^2^Department of Clinical Neurosciences and National Institute for Health Research (NIHR) Biomedical Research Centre, University of Cambridge, Cambridge, United Kingdom

**Keywords:** progressive multiple sclerosis, neural stem cell, regenerative neuroimmunology, mesenchymal stem cell, stem cell therapy, clinical trial

## Abstract

Multiple sclerosis (MS) is a chronic inflammatory disease of the central nervous system characterized by demyelination and axonal degeneration. MS patients typically present with a relapsing-remitting (RR) disease course, manifesting as sporadic attacks of neurological symptoms including ataxia, fatigue, and sensory impairment. While there are several effective disease-modifying therapies able to address the inflammatory relapses associated with RRMS, most patients will inevitably advance to a progressive disease course marked by a gradual and irreversible accrual of disabilities. Therapeutic intervention in progressive MS (PMS) suffers from a lack of well-characterized biological targets and, hence, a dearth of successful drugs. The few medications approved for the treatment of PMS are typically limited in their efficacy to *active* forms of the disease, have little impact on slowing degeneration, and fail to promote repair. In looking to address these unmet needs, the multifactorial therapeutic benefits of stem cell therapies are particularly compelling. Ostensibly providing neurotrophic support, immunomodulation and cell replacement, stem cell transplantation holds substantial promise in combatting the complex pathology of chronic neuroinflammation. Herein, we explore the current state of preclinical and clinical evidence supporting the use of stem cells in treating PMS and we discuss prospective hurdles impeding their translation into revolutionary regenerative medicines.

## Introduction

Multiple sclerosis (MS) is a chronic neuroinflammatory condition that affects over 2 million people worldwide (Stenager, 2019). The disease typically manifests in a relapsing-remitting (RR) form marked by sporadic attacks of neurological dysfunction (i.e., clinical relapses) followed by a (full or partial) functional recovery. While advances in the development of immunomodulatory disease-modifying therapies (DMTs) have had a substantial impact on the severity and frequency of relapses (Derfuss et al., 2020), within 30 years of diagnosis, two-thirds of RRMS patients will ultimately transition into the debilitating secondary progressive (SP) phase of the disease (Scalfari et al., 2014). During this phase, patients experience a gradual and ongoing accumulation of disability despite a lack of clinically evident relapses (Confavreux and Vukusic, 2014). In addition, 10–15% of MS patients present with a progressive form of the disease from the outset, a condition known as primary progressive (PP) MS.

Both forms of progressive MS (PMS) represent unmet clinical needs, as no available therapy is capable of arresting and repairing central nervous system (CNS) damage once progression ensues. Therefore, PMS therapeutic options (beyond conventional DMTs) should be devised to address the core drivers of this process to reduce chronic CNS compartmentalized neuroinflammation, enhance remyelination, and promote neural plasticity/regeneration.

In this sense, an extensive body of preclinical data supports the capacity of stem cell therapies to modulate the deleterious host immune responses and to facilitate neuroprotection in the CNS, which may be key to treat PMS.

## Understanding the Pathophysiology of PMS

The current success of DMTs mostly stems from their action on the adaptive immune system, a key driver of disease pathogenesis in the RR phase. Here, DMTs work to prevent acute inflammatory insults by limiting infiltration of activated T cells, B cells, and macrophages into the CNS where they contribute to the initial demyelinating insult that eventually leads to axonal loss and neurological disability (Mallucci et al., 2015). However, with increasing age and the subsequent transition of patients into the progressive phase of the disease, conventional DMTs no longer have a clear rationale of use nor provide obvious clinical benefits (Cunniffe et al., 2021). In fact, the progressive form of MS differs from RRMS in that it is a distinct neurodegenerative process shaped by persistent inflammation behind a *closed* blood-brain barrier (BBB) involving mostly activated microglia/macrophages, and only partially T and B cells.

**FIGURE 1:**
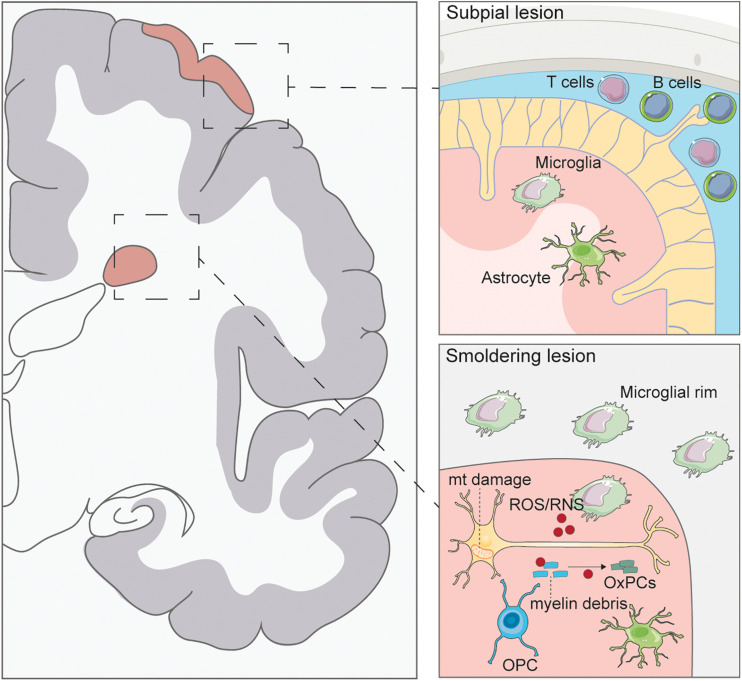
Pathology of progressive multiple sclerosis. Subpial lesions are typically found in PMS which are characterized by lymphocyte accumulation in the meninges. Activated T and B cells can secrete inflammatory cytokines causing microglia and astrocyte activation and ensuing demyelination of the cortex. Smoldering lesions are characterized by the degeneration of demyelinated neurons and surrounding microglial rim. Demyelinated axons have been found to have mitochondrial (mt) damage caused by ROS/RNS secretion from activated microglia. ROS/RNS can also oxidize myelin debris to generate oxidized phosphatidylcholines (OxPCs), which are toxic to neurons. Few oligodendrocyte progenitor cells (OPCs) are seen in these lesions, with no remyelination.

Understanding the pathological correlates of disease that contribute to the transition from RRMS to PMS and identifying dysfunctional mechanisms in PMS that lead to a persistent inflammatory disease state in the CNS is pivotal to identify new therapeutic strategies. Key mechanisms are discussed in the following sections and summarized graphically in Figure 1.

### The Role of Lymphocytes

The activation of lymphocytes and their infiltration into the CNS, which is typical of the RR forms of MS, is significantly decreased in PMS (Frischer et al., 2009). In early progressive disease, infiltration of lymphocytes (T and B cells) is compartmentalized at the leptomeninges and blood vessels of the CNS, beyond an intact BBB (Hochmeister et al., 2006). The extent of T cell infiltrate found within the meninges is directly correlated with the degree of axonal loss in the normal appearing white matter (NAWM) (Androdias et al., 2010; Lassmann, 2018). This implies that T cells, through the secretion of inflammatory factors, may contribute to CNS inflammation and damage. B cells have also been found to play a significant role in PMS by accumulating in the meninges and creating *de novo* structures called *ectopic follicles* (Serafini et al., 2004; Magliozzi et al., 2013). Here, B cells produce antibodies, secrete cytokines, and present antigens which further contribute to the persistence of inflammation (Aloisi and Pujol-Borrell, 2006). In fact, PMS patients presenting with follicle-like structures have a higher rate of disability and show a faster disease progression, associated with an increased lesion burden (Howell et al., 2011). This is thought to be at least partially the result of the extent and severity of meningeal inflammation in the formation of subpial cortical lesions (Howell et al., 2011; Choi et al., 2012). However, not all PMS patients present with inflammatory follicles, which suggests the presence of factors other than CNS B cells contributing to the persistent inflammation observed behind the intact BBB (Magliozzi et al., 2007). A number of studies have identified the presence of meningeal follicles only in SPMS and absent in PPMS (Magliozzi et al., 2007; Choi et al., 2012), yet a few others have identified follicular structures in PPMS cases associated with a rapid disease progression (Kutzelnigg et al., 2005; Haider et al., 2016; Cencioni et al., 2021).

### The Role of Mononuclear Phagocytes

In PMS, persistent tissue injury is associated with the activation of mononuclear phagocytes (MPs), consisting of both the CNS tissue resident microglia and the infiltrating monocytes that differentiate into macrophages (Lassmann et al., 2012). Interestingly, activation of MPs is found in many other neurodegenerative diseases without the associated pathological changes observed in MS (such as demyelination), suggesting that a specific mechanism may be required in MS to induce disease progression.

MPs in demyelinating lesions produce reactive oxygen species (ROS), reactive nitrogen species (RNS), and secrete pro-inflammatory cytokines and chemokines, including interleukin (IL)-1ß, IL-6, tumor necrosis factor (TNF)-α, and interferon (IFN)-γ, leading to oligodendrocyte and neuronal cell death. MPs are found in *smoldering* lesions, which are lesions unique to PMS patients that slowly expand due to clusters of activated MPs at the edge of these lesions (Reich et al., 2018). The continued activation and spreading of MPs in *smoldering* lesions contributes to further disease progression by increasing the size of the lesion and associated axonal damage (Kutzelnigg et al., 2005). Activated MPs, identified through their expression of CD68, are also found in the NAWM and normal appearing gray matter of patients with PMS. The number of activated MPs within the NAWM correlates with the extent of axonal injury, as measured by axonal swelling and degeneration, suggesting the neurodegenerative impact of diffuse MP inflammation (De Groot et al., 2001; Kutzelnigg et al., 2005; Absinta et al., 2019).

### The Role of Oligodendrocytes, Astrocytes and Neurons

Failure of the damaged CNS to regenerate and remyelinate is currently under intense study as the primary reason behind the transition from RRMS to PMS, since the presence of demyelinated axons in post-mortem neural tissue is highly prevalent during this stage of the disease (Franklin and Ffrench-Constant, 2008).

Findings from pre-clinical rodent models of MS have suggested that oligodendrocyte progenitor cells (OPCs) are functionally capable of remyelinating demyelinated axons (Franklin and Ffrench-Constant, 2017). However, recent findings from human studies have determined that the presence of myelinating oligodendrocytes in shadow plaques match the biological age of the patient. This suggests that myelinating oligodendrocytes within the plaque arise from the rearrangement of pre-existing oligodendrocytes rather than from newly generated OPCs (Yeung et al., 2019). Further work in humans using single cell RNA sequencing has revealed a depletion of OPCs in the NAWM of the PMS brain, implicating mature oligodendrocytes in early remyelination. However, significant differences in the transcriptome of mature oligodendrocyte populations were identified, including a decrease in intermediate oligodendrocytes and a skewed distribution of mature oligodendrocytes, suggesting a decreased regenerative potential (Jäkel et al., 2019). There remains significant debate as to whether OPCs are capable of remyelination in the human disease, if they are impeded by external and intrinsic factors, or if remyelination is primarily initiated by mature oligodendrocytes.

Astrocytes have been implicated in perpetuating CNS damage through secretion of inflammatory molecules, such as TNF-α and ROS that lead to both oligodendrocyte and neuronal cell death (Yi et al., 2019). In fact, the inhibition of astrocyte reactivity during experimental autoimmune encephalomyelitis (EAE), a mouse model of MS, ameliorates disease activity, suggesting a key role in neuroinflammation (Mayo et al., 2014). In chronic MS lesions, astrocytes maintain a hypertrophic response and form a glial scar in order to prevent spread of tissue destruction (Holley et al., 2003). However, the astrocytic scar can inhibit both remyelination and axonal regeneration, for example via the secretion of fibroblast growth factor-2 which promotes OPC proliferation but prevents differentiation (Goddard et al., 1999; Thümmler et al., 2019). Astrocytes have also been implicated in the production of hyaluronan, a glycosaminoglycan that accumulates in MS lesions, which can interact with CD44, a receptor found on neural stem cells (NSCs) (Pluchino et al., 2005), OPCs, astrocytes, and T cells (Sherman et al., 2002). The activation of CD44 on T cells induces proliferation and a cytokine response (Baaten et al., 2010), while treatment of OPCs with hyaluronan prevents their maturation into oligodendrocytes (Back et al., 2005).

Astrocytes play a key role in perpetuating inflammation in the MS CNS through the recruitment and activation of immune cells. The passage of leukocytes through the BBB is facilitated through increased expression of adhesion molecules such as vascular adhesion molecule (VCAM)-1 and intercellular adhesion molecule (ICAM)-1 on astrocytes. The expression of VCAM-1 on astrocytes is necessary for the entry and retention of T cells in the CNS parenchyma of EAE animals, as well as ensuing neurological disease (Gimenez et al., 2004). Further, the release of pro-inflammatory factors by astrocytes at the BBB such as IL-1ß, TNF-α, and chemokines C-C motif ligand 3 (CCL3) and C-X-C motif ligand 12 (CXCL12), attracts peripheral immune cells and increases permeability, thus allowing for their passage into the CNS (Minagar and Alexander, 2003; Calderon et al., 2006). Lastly, astrocytes affect the phenotype of T cells and microglial/macrophage activity in the CNS. Cytokines secreted by astrocytes promote the polarization of T cells and microglia/macrophages into pro-inflammatory states (Th1/Th17 and M1-like, respectively) (Saikali et al., 2010; Toft-Hansen et al., 2011; Zhou et al., 2011).

Neuronal damage is the key driver of brain atrophy, the prominent pathological feature of PMS (Bermel and Bakshi, 2006). Secondary, immune-mediated damage to neurons by peripheral lymphocytes is more common in the relapsing stage of the disease (Dutta and Trapp, 2011). On the other hand, primary neuronal damage is the key mechanism of damage in the progressive phase of the disease. Persistent demyelination of the axon – especially that driven by inflammatory factors such as ROS and cytokines – also renders the neuron more vulnerable to damage.

In PMS, neurons show impaired mitochondrial activity as evidenced by the decreased density of mitochondrial complexes I and III, resulting in a deficiency in the ability to generate energy to sustain normal cellular function (Friese et al., 2014). Mitochondrial DNA (mtDNA) deletions in genes that code for catalytic complexes’ subunits necessary for oxidative phosphorylation have been identified in PMS (Campbell et al., 2011), and analysis of common mtDNA sequence variations in MS populations identified a specific haplotype associated with an elevated risk of incurring mtDNA deletions in PMS (Tranah et al., 2015). The cumulative effect of mitochondrial abnormalities in neurons leads to the increased vulnerability of axons to external damaging stimuli contributing to their eventual degeneration. Interestingly, both brain atrophy and the number of transected axons correlate with the degree of inflammation in PMS lesions, suggesting a link between immune cell activation and neuronal damage (Frischer et al., 2009).

In this sense, ROS and RNS generated from both subsets of MPs may be the key drivers affecting mitochondrial functionality in neurons, thus leading to a highly destructive environment permissive to continued neuronal death.

### The Role of Cell Metabolism and Oxidative Stress

Metabolic signatures, such as differences in glycerophospholipids, have emerged as important readouts of PMS pathology and may aid in the diagnosis and the understanding of disease progression (Stoessel et al., 2018).

Several metabolites, or breakdown and intermediate products of cellular metabolism, are known to play important roles in regulating the inflammatory activity of immune and nervous system cells (Rothhammer et al., 2016). Succinate, an intermediate metabolite of the tricarboxylic acid cycle (TCA), increases and accumulates in the cerebrospinal fluid (CSF) of animals with EAE. Here, extracellular succinate exacerbates the pro-inflammatory activity of MPs, which further increased tissue damage (Peruzzotti-Jametti et al., 2018).

Clinically, analysis of CSF samples from MS patients has identified increased levels of lactate and altered levels of glucose in patients with PMS (Lynch et al., 1993; Simone et al., 1996). Follow-up untargeted mass spectrometry-based metabolomic studies have also identified alterations in lipid and energy metabolism in the CSF that are associated with a more severe disease progression in PMS patients. This may reflect the overall decrease in lipid content associated with increased demyelination (Villoslada et al., 2017).

Oxidized phosphatidylcholines (OxPCs) are another possible driver of neurodegeneration in PMS lesions. OxPCs are generated when myelin debris encounters free radicals leading to oxidized myelin. Prominent depositions of OxPCs are found in white matter lesions in PMS brains and associated with activated microglia. An *in vitro* study found OxPCs to be toxic to cultured neurons and oligodendroctyes (Dong et al., 2021). The combination of mitochondrial dysfunction, inflammatory metabolites (such as succinate) and inflammatory ROS/RNS may further generate OxPCs, thus contributing to the progression of MS pathology.

### The Role of Aging

Age is a prominent factor in the transition to PMS (Sanai et al., 2016; Scalfari et al., 2016). Several of the hallmarks that are associated with the aging process, including telomere attrition, mitochondrial dysfunction, cellular senescence, and stem cell exhaustion, have been linked to PMS (López-Otín et al., 2013; Oost et al., 2018). Cellular senescence is a biological process that can be induced via stress or replicative fatigue, triggering a variety of intrinsic cell processes including cell cycle arrest and secretion of a pro-inflammatory senescence associated secretory phenotype, which can have deleterious effects on the tissue microenvironment (Coppe et al., 2010).

Senescent progenitor cells are identified in lesions of PMS patients and associated with increased secretion of the pro-inflammatory alarmin high mobility group box 1 protein, which impairs OPC differentiation *in vitro* (Nicaise et al., 2019). Recent evidence further demonstrates that, with age, rodent OPCs are incapable of differentiating into mature oligodendrocytes which impairs their potential to regenerate lesioned areas (Neumann et al., 2019). Changes in OPCs due to aging and inflammation, such as DNA damage and reduced mitochondrial function, may account for the loss of remyelination in PMS.

Immunosenescence of macrophages and T and B cells could be another potential mechanism behind the decreased regenerative potential of the diseased CNS in PMS. Indeed, senescence of the immune compartment is understood to play a role in driving systemic aging in solid organs, including in immune privileged organs such as the brain (Yousefzadeh et al., 2021). Here, extracellular cholesterol generated from myelin breakdown overwhelms the phagocytic capability of aged macrophages and drastically impairs their ability to clear areas of inhibitory and damaging cellular debris (Cantuti-Castelvetri et al., 2018). T cell senescence has been observed in with RRMS and PMS patients, correlating perturbation of the immune system with age (Thewissen et al., 2005). Additionally, shorter telomeres, associated with senescent cells, have been identified in leukocytes of patients with PMS, with shorter telomere length correlated with increasing disability (Krysko et al., 2019).

Overall, a significant effect of aging is the increase in secreted inflammatory factors from senescent cells which can promote MS progression.

## Current and Emerging Therapies for PMS

### DMTs and Their Role in PMS

In RRMS DMTs are largely used to reduce and prevent relapses. IFN-ß and glatiramer acetate (GA) are immunomodulatory agents commonly recommended as first-line DMTs for RRMS. Other first-line DMTs for RRMS include teriflunomide and dimethyl fumarate (DMF), which are typically recommended as an option only if patients do not have a highly active or rapidly evolving severe disease. When these first-line DMTs are ineffective, the alternative is (1) switching to another first line DMT or (2) starting a second-line DMT (e.g., natalizumab, fingolimod, cladribine, alemtuzumab, or rituximab), which has greater efficacy but also more severe side-effects. This approach is defined as *escalation* therapy and is used for most RRMS patients.

In patients presenting with aggressive inflammatory disease at onset, consensus is that a more beneficial approach is to employ an *induction immune* therapy using second-line DMTs from the beginning of treatment (Roos et al., 2020), conferring a significantly lower risk of SPMS conversion (versus first-line *escalation* therapy) (Brown et al., 2019). Such an approach is more effective in reducing the risk of reaching a disability milestone, albeit with a worse safety profile (Prosperini et al., 2020).

Despite DMT treatment, the majority of RRMS patients will eventually experience a change in their MS, with fewer or no relapses over time but increasing disability and a decline in neurological function, reflecting an SPMS pattern (Inojosa et al., 2019). The transition from predominantly relapsing forms to more progressive forms of MS is gradual, and the two phenotypes inherently overlap for a period. In these *transitional* forms of MS, clinicians tend to continue the use of DMTs because of uncertainty in making a firm SPMS diagnosis, reluctance to stop treatment, and patients’ fear of disease activity returning upon withdrawal. However, the overall benefits of this approach are dubious, as many DMTs approved for RRMS showed negative or inconsistent results in clinical trials centered on SPMS patients (Fox et al., 2012). IFN-ß (Leary et al., 2003), GA (Wolinsky et al., 2007), fingolimod (clinicaltrials.gov identifier NCT00731692), and natalizumab (NCT01416181) (Kapoor et al., 2018) have all shown no clear efficacy in PMS patients.

Until recently, IFN-ß was the only DMT approved by the United Kingdom National Institute for Clinical Excellence (NICE) for people with SPMS, but only in the case of patients experiencing continuing relapses (i.e., *active* SPMS) (La Mantia et al., 2013). This recommendation came from evidence suggesting that IFN-ß reduced relapse risk in SPMS patients but was unable to significantly slow disability progression versus placebo (Panitch et al., 2004). The antineoplastic mitoxantrone was also approved as a potential therapy for SPMS by the US Food and Drug Administration (FDA), despite serious adverse effects related involving cardiotoxicity and therapy-related acute leukemia (Martinelli Boneschi et al., 2013).

Ocrelizumab (Ocrevus), a humanized anti-CD20 monoclonal antibody, was approved for PPMS patients by the FDA in 2017 and by NICE in 2019. The use of anti-CD20 antibodies stems from the initial observation that a single intravenous course of the anti-CD20 antibody rituximab reduces the inflammatory brain lesions in RRMS patients (Hauser et al., 2008). These data provided evidence of B-cell involvement in the pathophysiology of MS and prompted the use of anti-CD20 antibodies in PMS patients. Despite initial setbacks (Hawker et al., 2009), ocrelizumab was approved for patients with *active* PPMS thanks to the results of the ORATORIO study (Montalban et al., 2017); it is recommended for PPMS patients fulfilling specific clinical and radiological criteria consistent with *early active* disease.

In 2019, siponimod (Mayzent), a modulator of the sphingosine-1-phosphate (S1P) receptor (−1 and −5) (Gergely et al., 2012), was approved by the FDA as the first ever oral treatment for people with *active* SPMS, with NICE approval following the next year. Siponimod is a close structural analog of S1P, a naturally occurring bioactive sphingolipid that plays a key role in inflammation and repair processes. The S1P receptor is expressed by several CNS cells, including astrocytes, oligodendrocytes, neurons, microglia, and dendritic cells (Groves et al., 2013). By acting as a functional antagonist on S1P1 receptors on lymphocytes, siponimod prevents egress from lymph nodes, reducing the recirculation of T cells into the CNS to limit central inflammation. Moreover, siponimod can penetrate into the CNS and distribute into the white matter. Siponimod approval came after the results of the phase 3 EXPAND study (Kappos et al., 2018); it is recommended for treating *active* SPMS in adults.

Despite advances in PMS treatment, major hurdles still exist as these DMTs are limited to use in patients with an Expanded Disability Status Scale (EDSS) ≤ 6.5 due to lack of evidence in those with more severe disability. Moreover, no treatments are available for progressive patients who do not experience an *active* form of disease, making the identification of new therapies a key priority of MS research.

### Emerging Therapies for PMS

In recent decades several experimental or repurposed drugs have been tested in PMS but failed to advance past early phases of clinical testing due to a lack of efficacy (Ontaneda et al., 2017). These negative outcomes were disheartening, but also fostered the formation of several consortia aimed at identifying novel candidates for PMS treatment.

A recent combined systematic approach has reviewed existing evidence of human safety, BBB penetrance, demonstrable efficacy, and mechanistic targeting of licensed drugs for repurposing in PMS (Cunniffe et al., 2021). By focusing on processes and mechanisms of action that are specifically relevant to the pathogenesis of progression, four treatments were recommended for immediate testing in PMS: (R)-α-lipoic acid, metformin, the combination treatment of both (R)-α-lipoic acid and metformin, and niacin.

(R)-α-lipoic acid is a cofactor for at least five enzyme systems including pyruvate and α-ketoglutarate dehydrogenases, key enzymes of the TCA cycle. Results of a phase 2/3 trial in MS showed that treatment with lipoic acid induced a 68% reduction in annualized Percent Change Brain Volume while maintaining favorable safety, tolerability, and compliance over 2 years (Spain et al., 2017). Metformin, a synthetic derivative of guanidine commonly used as an oral antidiabetic, can reverse aging-associated remyelination failure, suggesting a possible application in PMS (Neumann et al., 2019). Niacin, a nicotinamide adenine dinucleotide precursor used for the treatment of hypercholesterolemia, has been shown to be protective against activated microglial-induced neurotoxicity and to promote oligodendrocyte proliferation *in vitro* (Kaneko et al., 2006). These mechanisms of action could be exploited in promoting regeneration and repair in PMS.

Besides these repurposed drugs, other therapies currently being tested in PMS include simvastatin, biotin, cladribrine, masitinib, ibudilast, and epigallocatechin-3-gallate (Faissner et al., 2019). Simvastatin has been studied in MS for its neuroprotective effects, which in part depend on the improvement of cerebrovascular hemodynamic (Neuhaus et al., 2004). A randomized, double-blind, placebo-controlled phase 2 clinical trial (MS-STAT) has shown that high dose simvastatin significantly reduces brain atrophy and radiological lesions in SPMS patients (Chataway et al., 2014). A larger phase 3 follow-up (MS-STAT2; NCT03387670) is now ongoing and will hopefully confirm these benefits. Biotin (vitamin B7) has been shown to (i) activate myelin formation in oligodendrocytes through its role as a cofactor for acetyl-CoA carboxylase, and to (ii) increase ATP production in axonal mitochondria, being a co-enzyme for three carboxylases (including the pyruvate carboxylase) of the TCA cycle (Sedel et al., 2015). The preliminary results of a phase 3 trial (NCT02220933) have shown that high dose daily administration has an impact on SPMS in reducing disease progression (Tourbah et al., 2016). A bigger cohort in a phase 3 clinical trial is currently being recruited. Cladribrine is approved for use in RRMS patients but a previous study found no significant treatment effects in terms of changes in EDSS scores in PMS (Rice et al., 2000). Nevertheless, cladribine produced significant sustained reductions in radiological lesion loads. These positive (but limited) outcomes sparked a new phase 3 trial, ChariotMS, looking to assess the beneficial role on cladribine on upper limb function in advanced PMS patients (EDSS between 6.5 and 8.5) (NCT04695080).

To summarize, therapies for PMS patients are beginning to emerge and hopefully we will experience a new era of therapeutics acting on the core drivers of disease progression. Most likely, successful therapeutic agents will have to interact with multiple processes, modifying chronic inflammation while enhancing the intrinsic repair of the damaged CNS. While more rigorous clinical trial design with appropriate endpoints and longer follow-up times may aid in the successful identification of safe and efficacious PMS DMTs (Huntemann et al., 2021), the innate multifunctionality of stem cell therapies offer a promising alternative route toward addressing the unmet needs of neuroprotection and neuroregeneration.

## The Basis of Stem Cell Therapies for the Treatment of PMS

Despite considerable success in treating RRMS, and a growing armamentarium of DMTs for combating *active* forms of PMS, there are substantial unmet needs for interventions capable of halting and reversing the chronic degeneration associated with PMS. In this light, there has been considerable interest in the presumed regenerative capabilities of stem cell therapies.

Stem cell therapy is a broad concept comprising the transplantation of different stem cell types sourced from various tissues into prospective patients for therapeutic effect. The choice of one cell type over another is based on multiple factors, but optimal outcomes will necessitate marrying appropriate mechanisms of action to the pathobiology being addressed.

### Hematopoietic Stem Cell Sources

A first important delineation in terms of therapeutic functionality exists between hematopoietic and non-hematopoietic therapies.

Hematopoietic stem cell (HSC) transplantation was the earliest cell therapy to emerge for the treatment of MS and it is currently the only clinically validated approach, having been imported from the field of hematology where it is routinely used in treating malignancies (Muraro et al., 2017a). HSC transplantation works by resetting the immune system by means of conventional immunoablation followed by reconstitution of the immune system by the stem cell graft. This results in the development of a novel immune system deprived of pathogenic auto-immune cells. Notably, whether the transplanted HSCs only provide means to overcome the cytopenia and toxicity caused by the immunosuppressive conditioning regimen, or whether there is a distinct transplant-associated anti-inflammatory effect, remains a matter of debate (Miller et al., 2021). Nonetheless, HSCs have little *regenerative* impact on the CNS as HSCs lack the ability to differentiate into neurons, astrocytes or oligodendrocytes (Gavriilaki et al., 2019). Thus, HSC transplantation is primarily efficacious for the treatment of clinical forms of MS with high inflammatory activity (i.e., RRMS or *active* PMS with clinical and/or radiological evidence of inflammation), but has limited efficiency in the case of inactive PMS, failing to address the degenerative component of the disease (Muraro et al., 2017a; Gavriilaki et al., 2019).

Consequently, *non-hematopoietic* stem cell (nHSC) transplantation has been embraced as a potential means to regenerate the damaged CNS in an attempt to offer a therapeutic solution for PMS.

### Non-hematopoietic Stem Cell Sources

While early studies of therapeutic nHSC transplantation typically sourced cells from embryonic or fetal tissue, the safety, practical, and ethical issues surrounding their prospective clinical use have caused current research efforts to shift toward the study of nHSCs derived from adult tissue (Hentze et al., 2009; Volarevic et al., 2018).

The most common nHSC types employed in preclinical and clinical studies of CNS disorders include: (1) mesenchymal stromal cells (MSCs), a heterogeneous class of multipotent cells derived from various tissues (Sharma et al., 2014; Hsuan et al., 2016; Martin et al., 2019); (2) bona fide neural stem cells (NSCs) derived from neurogenic brain niches (Boese et al., 2018); (3) pluripotent stem cell-derived NSCs, produced *ex vivo* through neural lineage differentiation of isolated embryonic stem cells (ESCs) (Cao et al., 2011; Tang et al., 2017; Oh et al., 2018; Zhao and Moore, 2018) or from differentiation of induced pluripotent stem cells (iPSCs), themselves generated by reprogramming of somatic cells such as fibroblasts acquired through a minimally invasive procedure (Takahashi and Yamanaka, 2006; Yu et al., 2007; Tang et al., 2017); and, most recently, (4) induced NSCs (iNSCs) obtained by *direct* reprogramming of a patient’s somatic cells, bypassing a pluripotent state (Kim et al., 2011; Han et al., 2012; Lujan et al., 2012; Thier et al., 2012; Daekee et al., 2019).

Mesenchymal stromal cells are a convenient source of nHSCs, being derived from various autologous or allogeneic tissues including bone marrow (BM-MSCs), adipose tissue (AD-MSCs), and the umbilical cord (UC-MSCs). While MSCs have also been reported to be able to differentiate toward non-mesodermal cells including neurons, astrocytes, and oligodendrocytes both *in vitro* and *in vivo*, their propensity for neural differentiation *in vivo* is limited (Wei et al., 2013; Squillaro et al., 2016). MSCs from different donors, various sources from the same donor, and even fractions of the same cell population are highly heterogenous, making it difficult to accurately establish their therapeutic efficacy (Bortolotti et al., 2015). Additionally, MSCs have been reported to exert immunosuppressive properties, raising concerns regarding patients potentially being at a greater risk of developing cancer due to the impaired surveillance activity of the immune system (Hasan et al., 2017).

Somatic NSCs possess several advantages for CNS applications over other stem cell sources, including their inherent commitment to the neural lineage, patient-specificity, and a low tumorigenic risk thanks to their lack of pluripotency and limited proliferation rate. However, the latter property comes at the expense of their low expandability *in vitro*, limiting the practicality of using somatic NSCs in large quantities. Additionally, extraction of NSCs from the neurogenic regions of the brain is difficult, invasive and carries significant risks (Nam et al., 2015). Indeed, human adult neurogenesis and the existence of NSC niches within the adult human brain has been a source of controversy in the scientific community (Lucassen et al., 2020). Instead, NSCs are almost exclusively sourced from fetal tissue, limiting their accessibility and raising ethical concerns.

These caveats led to the search for and identification of alternative sources of NSCs such as those obtained from ESCs or iPSCs, as well as iNSCs. These derived NSCs can recapitulate the properties, potency, and therapeutic potential of bona fide NSCs, making them ideal candidates to pursue regeneration of the CNS. Additionally, ESCs, iPSCs and iNSCs are readily expandable *in vitro*, and, in the case of iPSCs/iNSCs, autologous origins can minimize issues relating to immunogenicity, although not necessarily negating them completely (Wood et al., 2016). iNSCs offer a number of potential therapeutic advantages over pluripotent sources in that they are readily sourced, ostensibly easier, faster, and more cost-efficient to generate than iPSCs, and bypass a problematic pluripotency stage associated with tumorigenic risks (Erharter et al., 2019). In several cases, these multipotent sources have been further differentiated to specific neural lineages for study in a preclinical transplantation studies, with iPSC-derived oligodendrocyte progenitor cells (iPSC-OPCs) being a key example in the context of MS (Chanoumidou et al., 2020).

Nonetheless, it remains unclear to what extent autologous patient-derived NSCs may retain disease-specific epigenetic marks could hamper their therapeutic potential or have deleterious effects on other CNS cells (Nicaise et al., 2019).

### Mechanisms of Action for Non-hematopoietic Stem Cells

Non-hematopoietic stem cells exert their therapeutic function in a multifaceted fashion, targeting a broad range of deleterious disease processes, often in a tissue-specific manner, making them ideal candidates for treating diseases such as PMS with multiple overlapping pathological mechanisms.

**FIGURE 2:**
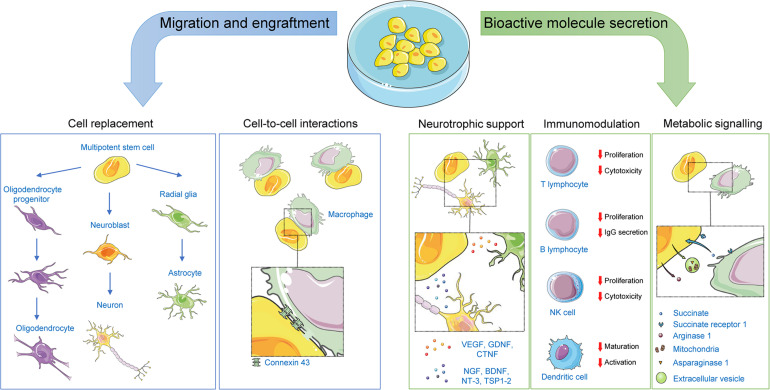
Mechanisms of action for non-hematopoietic stem cells. Following transplantation, non-hematopoietic stem cells can exert their therapeutic effects by: (1) replacing damaged CNS cells; (2) offering neurotrophic support to CNS cells via paracrine and juxtracrine signaling; (3) affecting immunomodulatory functions on both the innate and adaptative immune systems via paracrine and juxtracrine signaling, or via direct cell-to-cell contacts; and (4) engaging in metabolic signaling with cells within their niche. Representative examples of key players in each mechanism are illustrated. BDNF, brain-derived neurotrophic factor; CNTF, ciliary neurotrophic factor; GDNF, glial cell–derived neurotrophic factor; NGF, nerve growth factor; NT-3, neurotrophin-3; TSP1-2, thrombospondins 1 and 2; VEGF, vascular endothelial growth factor.

Originally, nHSC stem cell therapy was explored as a means to revert CNS damage by replacement of damaged cells by virtue of the self-renewal and potency properties of the graft. This view was supported by observations of successful engraftment and differentiation of stem cells into the CNS following transplantation in preclinical and clinical studies of CNS disorders. Indeed, multiple studies assessing stem cell engraftment and differentiation efficiency in several CNS conditions such as amyotrophic lateral sclerosis, spinal cord injury, and stroke have reported the successful synaptic integration of the graft and subsequent regeneration (Zhang et al., 2019). As touched upon below, transplanted nHSCs can also integrate *in vivo* without differentiation, instead exerting some of their therapeutic effects through mechanisms implying cell-to-cell interactions with the host. However, it is becoming increasingly clear that the therapeutic properties of nHSC transplantation extend well beyond differentiation, cell replacement and integration, factors shown to play only a secondary role in preclinical studies (Pluchino et al., 2005, 2009; Scolding et al., 2017). The currently accepted scientific view is that nHSC transplantation primarily exerts its beneficial effects by regulating the local environment through paracrine effects including trophic support, immunomodulation and metabolic signaling (Figure 2).

The neuroprotective and neurotrophic actions of nHSCs are directly exerted through the secretion of various factors (Willis et al., 2020). For example, one of the most clinically advanced NSC products, the CTX fetal NSC line, has been reported to secrete a complex cocktail of cytokines and growth factors that promote neurogenesis, axonal sprouting, and angiogenesis (Sinden et al., 2017). Likewise, it has been shown that MSCs secrete factors that induce axonal outgrowth and increase the survival of cells *in vitro* (Cho et al., 2009; Kim et al., 2010). Interestingly, MSCs have exhibited neuroprotective effects at a distance, without the need of their direct transplantation into the CNS, as a result of their capacity to secrete paracrine neurotrophic factors via extracellular vesicles (EVs) (Li et al., 2019; Mansoor et al., 2019). As membranous vectors of intercellular communication secreted by cells into the extracellular space, EVs are capable of influencing physiological and pathophysiological functions by trafficking bioactive cargoes such as proteins, lipids, and nucleic acids to recipient cells (van Niel et al., 2018; Xiao et al., 2021). EVs originating from various cell types are understood to influence the pathogenesis of MS and EAE (Dolcetti et al., 2020), highlighting their utility as biomarkers of disease. Conversely, exogenous stem cell-derived EVs act as delivery vehicles for prospective therapeutic factors (Wiklander et al., 2019), even demonstrating the ability to cross the BBB (Jan et al., 2017; Banks et al., 2020). On this basis, EV-based therapies are now being explored as a promising acellular alternative to stem cell transplantation, taking advantage of the stem cell secretome while minimizing disadvantages such as immunogenicity (Drago et al., 2013; Riazifar et al., 2017; Vogel et al., 2018; Branscome et al., 2020).

Mesenchymal stromal cells exhibit immunomodulatory functions via both direct and paracrine interaction with immune cells. Specifically, MSCs suppress T cell proliferation, inhibit the production of pro-inflammatory cytokines, and regulate the ratio of Th2/Th1 activated cells (Glennie et al., 2005; Puissant et al., 2005; Yanez et al., 2006). MSCs can also arrest B cell cycling and inhibit their division and antibody production (Corcione et al., 2006). Moreover, MSCs affect natural killer cells and dendritic cells by inhibiting their activation and maturation (Ramasamy et al., 2007; Burchell et al., 2010), and exert immunosuppressive activity by modulation of regulatory T cell function (Selmani et al., 2008). Several MSCs-derived soluble factors including transforming growth factor (TGF)-β1, prostaglandin E2 (PGE2), hepatocyte growth factor (HGF), indoleamine-pyrrole 2,3-dioxygenase, NO, and IL-10 have been proposed to mediate the immunosuppressive effects of MSCs (Gao et al., 2016). Importantly, previous studies showed that secretory factors vary greatly in relation to both the origin of MSCs and their environmental conditions (Mansoor et al., 2019).

Immunomodulatory functions of NSC transplantation have been primarily described to occur through juxtracrine and paracrine T cell signaling (Pluchino and Cossetti, 2013), as a result of their release of factors such as the cytokines IL-10 (Yang et al., 2009), leukemia inhibitory factor (LIF) (Cao et al., 2011), and TGF-β2 (De Feo et al., 2017), as well as NO and PGE2 (Wang et al., 2009). Consequently, NSC transplantation inhibits the peripheral and perivascular activation of proinflammatory T cells and increases the numbers of anti-inflammatory T regulatory cells (Tregs) in animal models of MS (Pluchino et al., 2005; Einstein et al., 2007). In addition to their paracrine functions, transplanted NSCs exert important anti-inflammatory effects on the adaptive immune system through cell-to-cell interactions, leading to reduced proliferation, decreased activation, and increased apoptosis of proinflammatory T lymphocytes (Pluchino et al., 2005; Fainstein et al., 2008). Transplanted NSCs have also demonstrated immunomodulatory properties through their direct interaction with local cells of the innate immune system, including macrophages (Cossetti et al., 2012). These observations are of particular importance for the treatment of PMS, when macrophages play a pivotal role in pathogenesis. Specifically, analysis of cell-to-cell interactions at the perivascular niches revealed the presence of tight contacts between NSCs and macrophages, established via connexin 43 (Cusimano et al., 2012).

Metabolic signaling has been described as a distinct mechanism by which stem cells mediate part of their immunomodulatory function. In local inflammatory microenvironments NSCs have been shown to engage in a homeostatic cell competition with MPs by changing their own metabolism and depleting the extracellular milieu of inflammatory immunometabolites such as succinate (Peruzzotti-Jametti et al., 2018). Additionally, cytokine-primed NSCs increase the secretion of extracellular arginase-1, inhibiting the proliferation of lymph node cells (Drago et al., 2016), and EVs derived from NSCs exposed to a pro-inflammatory stimulus were found to shuttle receptor-bound IFN-γ to recipient cells wherein it activates Stat1 signaling (Cossetti et al., 2014). NSCs-derived also harbor functional metabolic enzymes and thus act as independent metabolic units (Iraci et al., 2017). Conversely, addressing the chronic activation of mononuclear phagocytes by means of modulating their mitochondrial metabolism is expected to be a key target of future molecular and cellular therapies for PMS (Peruzzotti-Jametti and Pluchino, 2018).

A new phenomenon of mitochondrial transfer has been found to take place between MSCs and immune cells, modulating the function of the latter. Macrophages reportedly enhance their phagocytic capacity and bioenergetics profiles, and shift to an anti-inflammatory phenotype, following EV-mediated transfer of mitochondria from MSCs (Han et al., 2020). A similar phenomenon has been reported in NSCs, which release EV-trafficked mitochondria that are taken up by lipopolysaccharide-activated MPs and integrated into the host mitochondrial network, metabolically reprogramming the MPs and reducing their pro-inflammatory activation (Peruzzotti-Jametti et al., 2021).

Despite findings suggesting nHSCs as promising therapeutic tools for diseases such as PMS in which tissue repair is needed and/or inflammation is extensive, clinical translation of nHSC transplantation has met some barriers. These include limited engraftment duration, little *in vivo* differentiation, and restricted accessibility into damaged sites (Yousefi et al., 2019). Additionally, the complex functionality of stem cells makes it difficult to clearly correlate their distinct therapeutic mechanisms of action and resultant outcomes in clinical studies. In an attempt to increase the translational success of stem cell-based therapies, the use of cell-free stem cell products (i.e., stem cell-derived secretomes or extracellular vesicles) is currently being proposed as an alternative therapeutic strategy (Bai et al., 2012). The use of stem cell derivatives may circumvent many of hurdles of cell therapy including tumorigenicity, immune rejection, high costs, and time-consuming manufacturing. Moreover, there is increasing interest in engineering of stem cell lines to increase their production of paracrine regulators. For instance, NSC lines have been engineered to overexpress specific neurotrophic factors (Marsh and Blurton-Jones, 2017) while the neuroregulatory platform of the MSC-derived secretome has been modified by dynamic culturing of MSCs in computer-controlled bioreactors (Yousefi et al., 2019).

### Practical Considerations of Stem Cell Transplantation Therapy

The specific parameters regarding the optimal administration route, dose and timing for successful stem cell interventions are dictated by the cell type being administered and their mechanisms of action. Multiple prospective administration routes are available for CNS applications, each of which comes with its own pros and cons. The least invasive route is the intravenous (IV) injection (Wang et al., 2017). Of note, this systemic route is characterized by poor engraftment efficiency due to the entrapment of stem cells in organs including the lungs, spleen, bone marrow, and kidneys, preventing most cells from reaching the CNS parenchyma (Fischer et al., 2009; DePaul et al., 2015). Nevertheless, while IV injection of stem cells may not be the optimal method for direct cell replacement, it has instead the potential to be highly effective for cells which primarily exert their therapeutic effect through long distance paracrine modulation, such as MSCs. Alternatively, intraarterial injection of stem cells has been proposed to prevent the collection of cells in systemic organs and increase the incidence of cells reaching their CNS targets (Walczak et al., 2008; Argibay et al., 2017; Na Kim et al., 2017). However, caution should be taken when using this administration route as it runs the risk of causing microembolisms if the volume and concentration of injected cells are not carefully controlled (Argibay et al., 2017; Na Kim et al., 2017).

More invasive administration routes ranging from intrathecal (IT), intracerebroventricular (ICV), and even direct intraparenchymal (IP) injection have been proposed as efficient means of facilitating intracranial migration to sites of damage (Muir et al., 2011; Hurst et al., 2013; Donega et al., 2014). IP injection may not be a feasible option for PMS stem-cell transplantation because of the multicentric and/or diffuse nature of PMS lesions. Thus, administration of NSCs into the CSF by IT or ICV injection establishes a useful compromise between the invasiveness of the procedure and circumvention of biological barriers and may represent the best options with regards to the treatment of PMS (Scolding et al., 2017). Lastly, the transplantation of stem cells through the nasal cavity has been described as a novel non-invasive approach that ostensibly results in an effective migration into the CNS. However, the safety, migratory mechanisms, and overall efficacy of this procedure are still being studied, and additional reports are needed to properly assess the safety and reproducibly of this administration route (Li et al., 2015).

The optimal window for stem-cell administration is another key consideration to ensure the best clinical outcome. Specifically, while the early administration of nHSCs may prove beneficial as the graft counteracts neuroinflammation and provides neurotrophic support, the pro-inflammatory CNS environment which characterizes early RRMS and *active* forms of PMS can adversely affect cell engraftment, differentiation, and survival (Smith et al., 2020).

It is thus essential that the suitability of the recipient with respect to the clinical form of MS, the stage of progression, and the nature of the therapy being delivered are thoroughly assessed before stem cells are administered.

## Preclinical Evidence for Stem Cell Therapies in Treating PMS

While there is no perfect animal analog of human MS, various aspects of the disease (acute and chronic inflammation and demyelination) can be recapitulated across a variety of *in vivo* models (Procaccini et al., 2015; Burrows et al., 2019).

Preclinical evidence for the efficacy of stem cell therapy in PMS has relied heavily on EAE mouse models to recapitulate the immunologically driven tissue damage observed in PMS. EAE is typically induced through administration of myelin-derived antigens (proteolipid protein or myelin oligodendrocyte glycoprotein). Other methods commonly used to invoke MS-like pathology include cuprizone- or lysophosphatidylcholine-induced demyelination, JHM-strain murine hepatitis virus (JHMV)-caused encephalomyelitis and demyelination in susceptible rodents, and the shiverer model of congenital hypomyelination. Promising preclinical evidence of the safety and efficacy evidence of stem cell transplantation has been acquired with both HSC and nHSC sources, with a common theme of transplants resulting in immunomodulation, neuroprotection, and neurotrophic support in the chronically inflamed CNS. Below we briefly summarize key findings in this area, with a particular focus on nHSC experiments (as summarized in Table 1).

**TABLE 1:** Preclinical evidence of non-hematopoietic stem cells in treating MS.

Study	Cell Type/Source	Injection Route/Cell Dose	Timing	Animal Model of MS	Key Findings
**MSCs**					
**Zappia et al., 2005**	BM-MSCs of adult male WT mice	IV, 1 × 10^6^ cells	10, 15, or 24 dpi	MOG-induced chronic-EAE in WT female mice	• Improved EAE outcomes when transplanted before the chronic phase (before 24 dpi) • Grafted cells migrated and survived in the spinal cord and lymphoid organs • Decreased inflammatory infiltrates • Induction of T cell anergy
**Gerdoni et al., 2007**	BM-MSCs from adult WT mice	IV, 10^6^ cells	Onset of disease (ca. 12 dpi)	PLP-induced relapsing-remitting EAE in WT female mice	• Improved EAE outcomes with fewer relapses • Decreased inflammatory infiltrates, demyelination, and axonal loss • No evidence of graft integration in brain parenchyma • PLP-specific immune response reduced in MSC-treated mice
**Bai et al., 2009**	Human BM-MSCs	IV, 3 × 10^6^ cells	16 or 27 dpi	MOG-induced chronic-EAE and PLP-induced relapsing-remitting EAE in WT female mice	• Improved chronic and relapsing-remitting EAE outcomes • Survival and migration of grafted cells into demyelinating areas • Increased oligodendrocyte lineage cells surrounding lesions • Reduced inflammatory cell infiltration and demyelination in chronic EAE
**Constantin et al., 2009**	AD-MSCs from adult WT mice	IV, 1 × 10^6^ cells	3 and 8 dpi or 3 and 28 dpi	MOG-induced chronic EAE in WT female mice	• Improved EAE outcomes when transplanted before onset or chronically • Reduced demyelination and axonal loss • Shift toward Th2 T cell population • Survival of grafted cells and differentiation into OPCs
**Guo et al., 2013**	Human BM-MSCs	IV, 1 × 10^6^ cells	3 or 12 dpi	MOG-induced chronic EAE in WT female mice	• Improved EAE outcomes • Reduced inflammatory cell infiltration and demyelination • Increase in anti-inflammatory Th2 cells and anti-inflammatory cytokines • Lower serum levels of IL-6 and TNF-α
**Anderson et al., 2017**	AD-MSCs from adult WT male mice	IP, 1 × 10^6^ cells	At onset or the acute phase of disease (on the basis of clinical score)	MOG-induced chronic-EAE in WT female mice	• Improved EAE outcomes • Reduced inflammatory cell infiltration and demyelination • Reduced dendritic cell activation in the draining lymph nodes • Reduced autoantigen-specific T-cell function
**Xin et al., 2020**	BM-MSCs from adult WT mice	IP, 1 × 10^7^ cells	14 and 20 dpi	MOG-induced chronic-EAE in WT female mice	• Improved EAE outcomes • Shifted the polarization of macrophages from M1 to M2 • Inhibited the activation and proliferation of T cells
**Laso-García et al., 2018**	EVs from human AD-MSCs	IV, 25 μg EVs	60 days post-infection	TMEV-induced demyelination in WT female mice	• Improved motor function • Reduced brain atrophy • Decreased inflammatory infiltrates • Increased myelin expression • Reduced plasma levels of Th1/Th17 cytokines
**Jafarinia et al., 2020**	EVs from human AD-MSCs or human AD-MSCs	IV, 60 μg EVs or 1 × 10^6^ cells	10 dpi	MOG-induced chronic-EAE in WT female mice	• Improved EAE outcomes with EVs or cells • Decreased inflammatory infiltrates • Reduced demyelination
**Clark et al., 2019**	EVs from human placental MSCs or human placental MSCs	IV, 1 × 10^7^ or 1 × 10^10^ EVs, or 1 × 10^6^ cells	19 dpi	MOG-induced chronic-EAE in WT male and female mice	• Improved motor function outcomes with high-dose EVs or cells • Reduced oligodendroglial DNA damage • Increased myelination in the spinal cord
**Riazifar et al., 2019**	EVs from human BM-MSCs (native or IFN-γ) stimulated	IV, 150 μg EVs	18 dpi	MOG-induced chronic-EAE in WT female mice	• Improved EAE outcomes • Reduced demyelination • Decreased neuroinflammation • Upregulated Treg numbers
**Li et al., 2019**	EVs from rat BM-MSCs	IV, 150 or 400 μg EVs, or 1 × 10^6^ cells	On day of induction	Spinal cord homogenate-induced EAE in WT female mice	• Dose-responsive improved EAE outcomes with EVs or BM-MSCs • Decreased inflammatory infiltrates • Decreased demyelination • Increased expression of M2-like cytokines (IL-10, TGF-β) • Decreased expression of M1-like cytokines (TNF-α, IL-12)
**Hosseini Shamili et al., 2019**	EVs from mouse BM-MSCs	IV, 200 μg EVs or aptamer-modified EVs	1, 3, 6 dpi (prophylactic), or 12, 15, 18 dpi (therapeutic)	MOG-induced chronic-EAE in WT female mice	• Improved EAE outcomes by aptamer-modified EVs in prophylactic treatment regime • Decreased demyelination • Decreased inflammatory infiltrates
**NSCs**					
**Pluchino et al., 2003**	NSCs from SVZ of adult WT mice	ICV or IV, 1 × 10^6^ cells	10, 15, or 22 dpi	MOG-induced chronic-EAE in WT mice	• Improved EAE outcomes • Survival, integration, and neural differentiation of NSCs • Increased the quantity of OPCs • Reduced astrogliosis
**Pluchino et al., 2005**	NSCs from SVZ of adult WT mice	IV, 1 × 10^6^ cells	13 or 31 dpi	PLP-induced relapsing-remitting EAE in WT mice	• Improved EAE outcomes • Survival and integration of NSCs, but retention of immature stem-cell phenotype • Localization of grated NSCs around blood vessels • VLA-4 expression by NSCs • BMPs, noggin, notch-1, VEGF, and jagged-1 all upregulated
**Pluchino et al., 2009**	Human fetal NSCs	IV, 6 × 10^6^ cells, or IT, 2 × 10^6^ cells	Disease onset	Human MOG-induced EAE in common marmosets	• Improved EAE outcomes and increased survival • Effect more significant in IV-administered group • Graft survived undifferentiated for up to 3 months post-administration • Localization to perivascular inflammatory CNS regions and draining lymph nodes • Attenuated T cell proliferation and dendritic cell maturation
**Yang et al., 2009**	NSCs from SVZ of adult WT mice engineered to express IL-10	ICV or IV, 1.5 × 10^6^ cells	10, 22, or 30 dpi	MOG-induced chronic-EAE in WT mice	• Improved EAE outcomes when applied at onset, 10, 22, or 30 dpi • Differentiation into neurons and oligodendrocytes • Reduced demyelination, increased remyelination
**Peruzzotti-Jametti et al., 2018**	NSCs from SVZ of adult WT mice	ICV, 1 × 10^6^ cells	3 days post-onset (14–21 dpi)	MOG-induced chronic-EAE in WT female mice	• Improved EAE outcomes • Survival and integration of grafted cells • Accumulation within perivascular infiltrates • Reduction of extracellular succinate • Reprogramming of immune cells toward anti-inflammatory phenotype • Reduced demyelination and axonal loss
**ESCs**					
**Aharonowiz et al., 2008**	Human ESC-NSCs	ICV, 5 × 10^5^ cells	10 dpi	MOG-induced chronic-EAE in WT female mice	• Improved EAE outcomes • Reduced axonal damage and demyelination • Reduction of encephalitogenic T cells
**Cao et al., 2011**	Mouse ESC-NSC	IV, 2 × 10^6^ cells	0 or 10 dpi	MOG-induced chronic-EAE in WT mice; sex not reported	• When administered 0 dpi, disease onset was delayed, symptoms were reduced, inflammation and demyelination were decreased • When administered 10 dpi, ameliorated EAE symptoms, inhibited proliferation and cytokine production of T cells via LIF
**Chen et al., 2014**	Human ESC-NSCs	Intraspinal, 2.5 × 10^5^ cells	14 dpi	JHMV-induced encephalomyelitis; sex not reported	• Improved functional outcomes • Reduced neuroinflammation • Reduced accumulation of CD5^+^ T cells • Increase in regulatory T cell populations • Decreased demyelination and enhanced remyelination
**iPSCs**					
**Laterza et al., 2013**	iPSC-NSCs from MEFs	IT, 1 × 10^6^ cells	4 days post-onset (dpi not reported)	MOG-induced chronic-EAE in WT female mice	• Improved EAE outcomes through neuroprotection, not cell replacement • Survival of grafted cells and accumulation within perivascular infiltrates • Reduced demyelination, enhanced remyelination via activation of LIF pathway
**Thiruvalluvan et al., 2016**	Human iPSCs-OPCs from human dermal fibroblasts	Injection in the corpus callosum, 5 × 10^4^ cells	79 dpi in marmosets, at onset in mice (dpi not reported)	MOG-induced EAE in female common marmosets; cuprizone in female mice; MOG-induced chronic-EAE in WT female mice	• Survival and migration of grafted cells to CNS lesions in EAE marmosets; differentiation into mature and myelin-forming oligodendrocytes in EAE marmosets • Improved EAE outcomes in mice through reduced inflammatory cell infiltration and demyelination in EAE mice, but no cell engraftment • Grafted cell survival in cuprizone mice and partial differentiation into mature oligodendrocytes
**Zhang et al., 2016**	iPSC-NSC from MEFs	ICV, 2 × 10^5^ cells	18 dpi	MOG-induced chronic-EAE in WT male mice	• Improved EAE outcomes • Reduction of T cell infiltration • Ameliorated white matter damage
**iNSCs**					
**Lujan et al., 2012**	iNSCs from MEFs	Intracerebellar, 1 × 10^5^ cells	1 day postnatal	Shiverer mouse model of congenital hypomyelination	• Remyelination in the white matter tracts of the cerebellum
**Peruzzotti-Jametti et al., 2018**	iNSCs from MEFs	ICV, 1 × 10^6^ cells	3 days post-onset (14–21 dpi)	MOG-induced chronic-EAE in WT female mice	• Improved EAE outcomes • Survival and integration of grafted cells • Accumulation within perivascular infiltrates • Reduction of extracellular succinate • Reprogramming of immune cells toward anti-inflammatory phenotype • Reduced demyelination and axonal loss
**Sullivan et al., 2020**	iNSCs from MEFs	Corpus callosum injection, 1 × 10^4^ cells	12-week cuprizone diet	Cuprizone-induced chronic demyelination in WT male mice	• Astroglial and oligodendroglial differentiation; many undifferentiated • Demyelination unaffected but increased endogenous oligodendrocytes and proliferating OPCs • Reduced astrogliosis • Amelioration of motor deficits

*BMP, bone morphogenic protein; CNS, central nervous system; dpi, days post-immunization; EAE, experimental autoimmune encephalomyelitis; ESC, embryonic stem cell; ICV, intracerebroventricular; IL, interleukin; iNSC, induced neural stem cell; IP, intraperitoneal; iPSC, induced pluripotent stem cell; IV, intravenous; JHMV, JHM-strain murine hepatitis virus; LIF, leukemia inhibitory factor; MEF, mouse embryonic fibroblast; MOG, myelin oligodendrocyte glycoprotein; MSC, mesenchymal stromal cell; NSC, neural stem cell; OPC, oligodendrocyte progenitor cell; PLP, proteolipid protein; SUCNR1, succinate receptor 1; SVZ, subventricular zone; TMEV, Theiler’s murine encephalomyelitis virus; TNF, tumor necrosis factor; VEGF, vascular endothelial growth factor; WT, wild-type.*

### Preclinical Evidence for HSCs

Immunoablation and subsequent reconstitution by means of autologous HSC transplantation (HSCT) is now seen as a viable clinical treatment option for *early active* MS (see section “Clinical Studies of Stem Cell Therapies in Progressive Multiple Sclerosis”), with a strong body of supporting preclinical data from EAE studies extending back several decades (van Bekkum, 2004; Van Wijmeersch et al., 2008).

In key supporting *in vivo* rodent experiments, the effectiveness of post-conditioning syngeneic HSCT (in the form of a bone marrow transplant) in combatting EAE was found to be highly dependent upon the timing of the intervention, with early treatment having substantial preventative outcomes (Karussis et al., 1992; Karussis et al., 1993) but little effect during the chronic stages of EAE (Karussis et al., 1993; van Gelder et al., 1993; Burt et al., 1998; Herrmann et al., 2005). Bone marrow transplant during the peak of disease greatly reduced spontaneous relapse rates (van Gelder et al., 1993), while a study of non-myeloablative conditioning in mice suggests the long-term EAE remission is dependent on a transplant-associated induction of regulatory T cells (Meng et al., 2011). Notably, pseudo-autologous transplants, obtained from syngeneic bone marrow donor mice in the same stage of EAE as the recipient, were found to be associated with higher levels of induced relapses than typical syngeneic treatments, despite similar efficacy with regards to disease prevention (van Gelder and van Bekkum, 1996).

Allogeneic transplants have typically been found more effective than syngeneic or pseudo-autologous sources in preventing relapses in EAE rodents (van Gelder and van Bekkum, 1995, 1996). There may be implications here with regards to the optimal choice of allogeneic versus autologous HSC sources for therapeutic use, however, clinical HSCT routinely employs autologous sources, backed by assuring efficacy and safety evidence (Alexander et al., 2021; Miller et al., 2021).

### Preclinical Evidence for MSCs

Mesenchymal stromal cells have seen extensive study across multiple disease models, including CNS disorders, as they are an easily accessible source of autologous or allogeneic somatic stem cells with the capacity to differentiate into multiple lineages including mesodermal, ectodermic, and endodermic cells (Gugliandolo et al., 2020). However, the limited ability of MSCs to differentiate *in vivo* (Nombela-Arrieta et al., 2011) means that the therapeutic effects of MSCs stem largely from their paracrine effects, secreting cytokines, growth factors, small RNAs, and EVs (Gugliandolo et al., 2020). The IV injection of BM-MSCs in mouse models of EAE at the onset (10 days post-immunization [dpi]) and peak (15 dpi) of disease, but not during the chronic phase (24 dpi), improves functional outcomes, decreases inflammatory cell infiltration, induces T cell anergy, and suppresses pathogenic B cells (Zappia et al., 2005; Gerdoni et al., 2007). Further evidence for the immunomodulatory role of BM-MSCs arises from a study where MSCs were transplanted intraperitoneally at the peak (14 dpi) and again at the chronic (20 dpi) stages of EAE in mice (Xin et al., 2020). Amelioration of clinical scores was observed, correlating with reduced CD4^+^ and CD8^+^ T cell activation and proliferation, increased numbers of Tregs in the spleen, and a shift in the polarization of macrophages from M1 to M2, ultimately reducing the splenic production of pro-inflammatory cytokines, IFN-γ and IL-17. Despite these positive outcomes, transplanted cells were rarely observed in the CNS parenchyma, in line with notion that the therapeutic effects of MSCs transplants arise primarily through paracrine mechanisms taking place peripherally.

Nonetheless, cases of neural differentiation and integration have been reported. IV injected AD-MSCs successfully integrated into the spinal cord of mice with EAE at 3 months; most cells had differentiated into OPCs, but a limited differentiation into astrocytes or mature oligodendrocytes was also observed (Constantin et al., 2009). When administered before EAE onset (3 and 8 dpi) AD-MSCs improved functional outcomes and reduced inflammation. When administered in the chronic phase (23 and 28 dpi) functional deficits were again significantly ameliorated, endogenous OPCs were increased around the demyelinating lesions, and the number of anti-inflammatory Th2 cells was increased (Constantin et al., 2009). In a similar study, AD-MSCs sourced from either mice or humans were transplanted intraperitoneally at onset or during the acute phase of EAE and, in both cases, clinical scores were improved (Anderson et al., 2017). Further *in vitro* and *in vivo* investigation revealed that in mice with EAE, the transplantation of syngeneic AD-MSCs improved symptoms by reducing both autoantigen-specific T cell function and the activation of dendritic cells in draining lymph nodes.

Xenografts of human MSCs in mouse models of EAE have had similar success when compared to their mouse counterparts. The IV transplantation of human BM-MSCs at the peak (16 dpi) or chronic stage (27 dpi) of EAE improved functional outcomes and reduced inflammatory cell infiltration and demyelination (Bai et al., 2009). In contrast to previous findings from mouse MSC grafts (Zappia et al., 2005; Gerdoni et al., 2007), human BM-MSCs migrated to the CNS within 24 h, where they persisted for up to 45 days (albeit with decreasing numbers). Similarly, the IV transplantation of human BM-MSCs at 3 dpi or at onset (12 dpi) improved functional outcomes and reduced inflammatory cell infiltration, including pro-inflammatory Th1 and Th17 cells, while increasing the number of anti-inflammatory Th2 cells and levels of anti-inflammatory cytokines (Guo et al., 2013). Furthermore, greater quantities of oligodendrocyte lineage cells were observed surrounding lesions.

Beyond the cells themselves, MSC-derived EVs have received considerable attention as a prospective MS therapy. Human AD-MSC EVs, when delivered IV to Theiler’s murine encephalomyelitis virus-induced demyelinating disease mice (Laso-García et al., 2018) or EAE mice (Jafarinia et al., 2020), were found to improve functional outcomes and decrease inflammatory infiltrates, with evidence of reduced demyelination in the latter model. Likewise, upon IV administration at peak mouse EAE, human placental MSC-derived EVs yielded improved motor function and myelination (Clark et al., 2019) while EVs from IFN-γ stimulated human BM-MSCs increased Treg cell numbers with a concomitant decrease in neuroinflammation and demyelination (Riazifar et al., 2019). Early/prophylactic IV administration of rat BM-MSC EVs, when utilized in a spinal cord homogenate-induced rat EAE model, were found to induce a substantial polarization of CNS microglia toward an anti-inflammatory M2-like state, with improved behavioral outcomes and decreased inflammation and demyelination (Li et al., 2019). *Ex vivo* functionalization of EVs is also a promising aspect of this acellular approach, with, for example, mouse BM-MSC derived EVs adorned with a myelin-binding aptamer found to suppress inflammation and demyelination in EAE mice (Hosseini Shamili et al., 2019). The aptamer-modified EVs yielded improved clinical scores compared to the non-modified EVs when employed prophylactically. While EVs of various sources are under investigation as a distinct therapeutic intervention in several clinical trials, none are yet being conducted in the context of MS.

Extensive preclinical evidence supporting the paracrine and immunomodulatory effects of MSCs (or their acellular products) in preclinical models of MS, as well as safety and feasibility data from clinical study in other disease contexts, has seen their adoption as the main nHSC source for clinical trials to date.

### Preclinical Evidence for NSCs

As self-renewing, multipotent cells capable of differentiating into functional neurons and glial cells, NSCs are apposite to therapeutic applications in the CNS. Small populations of NSCs are found in the subcortical white matter (Nunes et al., 2003) and the regions of adult neurogenesis (subventricular zone (SVZ) and sub-granular zone of the hippocampus), and the discovery that proliferating NSCs can be maintained in culture (Reynolds and Weiss, 1992) inspired the study of NSC transplantation as a therapeutic approach for CNS pathologies, including PMS.

In the context of EAE, the IV and ICV transplantation of SVZ NSCs before disease onset (10 dpi), at onset (15 dpi), and the peak of disease (22 dpi) have ameliorated functional deficiencies in mice. Transplanted NSCs localized to areas of brain with demyelination and axonal loss within one-month post-transplantation (Pluchino et al., 2003), with migration to the lesion via chemotaxis reliant on the expression of very late antigen-4 (VLA-4) adhesion molecules and the activation of G-coupled protein receptors (Pluchino et al., 2005). Histopathological analysis demonstrated that a significant fraction of transplanted NSCs differentiated into OPCs, and ultimately reduced glial scarring at the lesion sites. Additionally, the IV transplantation of NSCs during chronic EAE also improved functional outcomes by promoting apoptosis of pro-inflammatory T-cells and reducing inflammatory immune cell infiltrate (Pluchino et al., 2005). To further enhance the anti-inflammatory effects of NSC transplants in EAE, NSCs from the SVZ were genetically engineered to secrete the anti-inflammatory cytokine IL-10 (Yang et al., 2009). When transplanted IV or ICV before disease onset (10 dpi), at the peak of disease (22 dpi), or during the chronic phase (30 dpi), grafted IL-10-secreting NSCs were found to amplify the therapeutic effects observed with control NSC transplants (Yang et al., 2009). The therapeutic properties of human NSCs have also been demonstrated in non-human primate models of EAE, where decreased disease severity and improved functional outcomes were observed in both IT- and IV-injected animals, with the IV-treated cohort demonstrating the greater improvement along with a substantial survival benefit (Pluchino et al., 2009). Human NSC xenografts survived undifferentiated for up to 3 months after administration, distributing to perivascular inflammatory CNS regions and attenuating T cell proliferation and dendritic cell maturation.

The initial expectation of NSC therapeutics was differentiation into neural cells and incorporation into the damaged CNS. However, it has become clear from more recent preclinical studies that these outcomes are secondary to the effects of immunomodulation and the promotion of neuroprotection and homeostasis (Smith et al., 2020), the so-called ‘*bystander effect,’* with NSCs possessed of a therapeutic plasticity allowing them to respond to endogenous (patho)physiological stimuli (Martino and Pluchino, 2006). In the context of PMS, the immunomodulation of MPs by transplanted NSCs is very promising. Preclinical evidence from mouse models of EAE suggests that NSCs can alter the proinflammatory phenotypes of MPs by sequestering the extracellular immunometabolite succinate and secreting anti-inflammatory PGE2 in response (Peruzzotti-Jametti et al., 2018). Transplanted NSCs home to meningeal perivascular areas where they localize in close contact with MPs, which in turn undergo metabolic reprogramming toward anti-inflammatory oxidative phosphorylation, with a resultant amelioration of chronic neuroinflammation and functional recovery in EAE mice. Notably, similar results were found using both somatic NSCs and iNSCs (Peruzzotti-Jametti et al., 2018).

Given these promising findings but otherwise limited accessibility of NSCs, there is a demand for more practical sources of NSCs to support clinical study, thus inspiring preclinical investigations of ESCs, iPSCs, and iNSCs.

### Preclinical Evidence for ESC-Derived Cells

Embryonic stem cells are pluripotent cells derived from the inner cell mass of the blastocyst and – of interest to MS therapeutic applications – they can be differentiated into NSCs or OPCs (Piao et al., 2015; Zhao and Moore, 2018; Chanoumidou et al., 2020). An IV injection of ESC-NSCs on the day of EAE immunization delayed the onset of disease in mice, reduced clinical scores, and decreased both inflammation and demyelination (Cao et al., 2011). When administered at 10 dpi, the transplants still ameliorated EAE symptoms, inhibiting the proliferation and cytokine production of T cells via the secretion of LIF.

As observed with MSCs, xenografts of human ESC-NSCs yielded similar results. Human ESC-NSCs transplanted ICV before disease onset (10 dpi) in mouse models of EAE reduced axonal damage and demyelination, decreased the quantity of encephalitogenic T cells, and ultimately improved clinical scores (Aharonowiz et al., 2008). While transplanted human ESC-NSCs were found to migrate to the brain parenchyma, differentiation into mature oligodendrocytes was not observed and the extent of remyelination was negligible. Similarly, in JHMV mouse models of MS, intraspinal injection of human ESC-NSCs were found to survive in the spinal cord parenchyma for only 1 week post-transplantation, but through immunomodulatory and paracrine effects the cells improved functional outcomes, reduced demyelination/increased remyelination, and decreased neuroinflammation with increased CD4^+^CD25^+^FOXP3^+^ Tregs and depleted CD5^+^ T cells in the spinal cord (Chen et al., 2014). *In vitro* studies found that human ESC-NSCs reduced T cell proliferation and, as observed *in vivo*, increased the number of Tregs.

Although ESC-derived NSCs have shown promising preclinical results, their use is burdened with ethical concerns over the source of the cells, as the collection of ESCs destroys the donor blastocyte. Furthermore, residual pluripotency from contaminating undifferentiated ESCs remains a safety issue. Thus, alternative non-ESC sources are favored for future clinical applications.

### Preclinical Evidence for iPSC-Derived Cells

Preclinical evidence for the feasibility of mouse iPSC-derived cells for autologous transplantation in MS was first demonstrated in EAE (Laterza et al., 2013). At the peak of disease, iPSC-derived NSCs were transplanted ICV into the cisterna magna, resulting in reduced demyelination and axonal damage, lower quantities of infiltrating inflammatory cells, improved functional outcomes, and the activation of the LIF pathway, as described for ESC-NSCs (Cao et al., 2011). Neither toxicity nor tumorigenicity was observed (Laterza et al., 2013). Furthermore, iPSC-NSCs displayed homing effects like those of somatic NSCs (Pluchino et al., 2003), and were found to localize to either demyelinating lesions or sites of increased inflammatory cell infiltration. In line with the *bystander* hypothesis, most of the transplanted cells did not differentiate and replace damaged neural cells, but rather their regenerative effects stemmed from the iPSC-NSC secretion of LIF, a neuroprotective, trophic cytokine that promotes endogenous OPC and oligodendrocyte growth (Laterza et al., 2013). Similarly, a later study performed ICV transplants of iPSC-NSCs at peak of disease (18 dpi) and observed amelioration of EAE symptoms and decreased T cell infiltration (Zhang et al., 2016).

Induced pluripotent stem cells can be differentiated into specific neural cell types *in vitro*, including oligodendrocytes or OPCs. In PMS there is a loss of endogenous OPC functionality and thus attempts have been made to replace these cells with pre-differentiated iPSC-OPC transplants. When transplanted ICV into marmoset and mouse models of EAE, iPSC-OPCs were shown to decrease inflammatory cell infiltration, reduce demyelination, and improve functional outcomes through bystander effects, with minimal transplanted cells surviving within the CNS parenchyma. However, if transplanted directly into the parenchyma, histopathological analysis revealed that the majority of iPSC-OPCs differentiated into mature oligodendrocytes capable of remyelinating axons, as observed via electron microscopy, while the remainder retained their OPC characteristics or differentiated into astrocytes (Thiruvalluvan et al., 2016).

Nevertheless, iPSC technology faces a number of hurdles to be addressed before successful clinical translation. There is growing evidence that the epigenetic signature of the donor cell can be maintained after iPSC induction. This can lead to issues of immune rejection of the transplants (Zhao et al., 2011), or unexpected iPSC functions. In one example, iPSC-NSCs generated from blood samples of PPMS patients lacked the neuroprotective phenotype observed in control iPSC-NSCs when transplanted into cuprizone-induced mouse models of demyelination (Nicaise et al., 2017). Furthermore, the two-step induction process required to generate iPSC derived neural cells and the expansion needed to produce sufficient cell counts for transplants is very lengthy, increasing the chance of genetic instability leading to tumorigenicity upon transplantation (Lee et al., 2013).

Thus, despite potentially exciting *in vivo* results, iPSCs and iPSC-derived neural cells still face substantial barriers to clinical translation.

### Preclinical Evidence for iNSCs

In recent years iNSC technology has emerged as a promising stem cell-based approach to CNS regeneration. iNSCs can be directly transdifferentiated from somatic cells such as fibroblasts, bypassing a potentially hazardous pluripotency stage and, in the case of autologous transplants, largely circumventing immunogenicity concerns.

Several studies have shown that iNSCs can be stably expanded *in vitro* and, like NSCs, secrete pro-regenerative molecules such as glia cell-derived neurotrophic factor (GDNF) and brain derived neurotrophic factor (BDNF) (Gao et al., 2017). Moreover, transplanted iNSCs have demonstrated long-term functional integration into the CNS *in vivo*, highlighting their potential for regenerative applications (Hemmer et al., 2014).

In shiverer mouse models of dysmyelination, iNSCs transplanted into the cerebellum of post-natal day 1 pups were able to differentiate, albeit slowly over 10 weeks, into functional oligodendrocytes capable of myelination (Lujan et al., 2012). Similarly, iNSCs grafted into the chronically demyelinated corpus callosum of cuprizone-treated mice were found to differentiate along oligodendrocyte and astrocyte lineages, although many remained undifferentiated (Sullivan et al., 2020). While iNSC transplantation was not found to mitigate demyelination in this model, endogenous oligodendrocytes and proliferating oligodendrocyte progenitors were increased in iNSC recipients. Notably, astrogliosis was significantly reduced in mice receiving transplanted iNSCs, with a concomitant amelioration of motor deficits (Sullivan et al., 2020). In the more PMS-relevant model of EAE, iNSCs transplanted ICV were found to be therapeutically equivalent to their somatic NSC counterparts, migrating to meningeal perivascular areas of EAE-affected mice where they reprogrammed pro-inflammatory MPs with resulting amelioration of chronic neuroinflammation and associated behavioral deficits (Peruzzotti-Jametti et al., 2018).

Overall, several putative stem cell therapies have demonstrated compelling preclinical safety and efficacy data in the context of MS models, with a consensus on the beneficial bystander/paracrine effects of the transplants, including during more chronic timepoints. Going forward, further characterization of the mechanisms of action, in support of optimized administration variables such as the timing, route, and dose of cells delivered, will be invaluable in translating preclinical evidence into clinical study.

## Clinical Studies of Stem Cell Therapies in Progressive Multiple Sclerosis

Despite a wealth of preclinical data supporting the potential of cell therapies in treating neuroinflammatory conditions such as MS, definitive clinical evidence is lacking (Smith et al., 2020). While the last two decades have seen numerous early phase clinical trials of putative stem cell therapies in treating MS, with compelling evidence of the safety and feasibility of the approach, there is a strong need for more robust efficacy data to assert the translational potential of the approach. Most published clinical trial data relates to small safety and tolerability studies not statistically powered or appropriately controlled to infer therapeutic benefit, while the heterogeneity of larger studies has made it difficult to measure the efficaciousness of specific interventions. Reported trials exhibit variability in a diversity of parameters, including the makeup of the patient cohort, trial design, and nature of the therapy (Smith et al., 2020). While suggestions of beneficial outcomes often emerge from these clinical studies, larger and/or more rigorous studies are needed to discern signal from noise before we can gauge the potential impact of emerging stem cell therapies on the lives of PMS patients (Pluchino et al., 2020). The following is an overview of clinical studies in support of the therapeutic use of stem cell therapies, with a particular focus on nHSC interventions (summarized in Supplementary Table 1).

### Clinical Evidence for HSCs in PMS

Some of the earliest clinical evidence of the utility of cell therapy in treating MS has arisen serendipitously, with reports of MS patients experiencing clinical improvements after undergoing immunoablation and allogeneic bone marrow transplantation for the treatment of concomitant leukemic or lymphoid malignancies (van Bekkum et al., 1996; McAllister et al., 1997).

Chemotherapeutic depletion of autoreactive immune cells followed by reconstitution through HSC transplantation (HSCT, employing bone marrow, peripheral blood, or umbilical cord blood sources) has been undergoing clinical study for several decades (Bakhuraysah et al., 2016; Muraro et al., 2017a; Oliveira et al., 2021), including in patients with PMS (Fassas et al., 1997), with beneficial functional outcomes commonly associated with the treatment. While intervention-associated morbidity and mortality has decreased substantially since the earliest reports (Snowden et al., 2017; Das et al., 2019), there remain ongoing efforts to delineate the optimal compromise between efficacy and safety with regards to the immunoablative conditioning regime (Das et al., 2019; Miller et al., 2021). Autologous HSCT is preferred over allogeneic transplants due to a significantly higher risk of adverse events such as graft-versus-host disease in the latter (Lu et al., 2010), as well as a lack of supporting clinical data for allogeneic grafts (Sharrack et al., 2020).

Evidence from long-term clinical studies points to beneficial effects of autologous HSCT in impeding disability worsening over the 5–10 years post-transplant (Muraro et al., 2017b; Boffa et al., 2021) with an impact exceeding that of DMTs (Muraro et al., 2017a), however, the efficacy of the treatment appears to favor younger patients, those with RRMS, and those with lower disability scores. On the recommendations of groups such as the (United States) National Multiple Sclerosis Society (Miller et al., 2021) and European Society for Blood and Marrow Transplantation (Sharrack et al., 2020), autologous HSCT may be beneficial for the treatment of *active* RRMS that is refractive to current DMTs (or in cases wherein these are contraindicated). Indeed, some appraisals suggests that HSCT may represent a cost-effective alternative to DMTs for the treatment of RRMS (Burt et al., 2020), however, larger studies are ongoing to ascertain whether HSCT is truly advantageous to current best standards of care [e.g., (Burt et al., 2019; Zhukovsky et al., 2021), and the BEAT-MS trial, NCT04047628]. While promising for the treatment of RRMS, the weight of clinical evidence suggests that autologous HSCT has limited efficacy in treating chronic PMS without superimposed disease activity (Bowen et al., 2012; Miller et al., 2021).

This observation supports the concept of a switch from an adaptive immune response mediated by infiltrating lymphoid cells during *active/relapsing* MS toward a compartmentalized CNS inflammation with a substantial (non-hematopoietic) MP-driven innate immunity component during PMS (Pluchino et al., 2020).

### Clinical Evidence for MSCs in PMS

In terms of a putative “*regenerative*” therapies, non-hematopoietic cell sources are typically employed. MSCs have received the most attention, both with regards to applications in MS (Oliveira et al., 2019) and more generally (Tavakoli et al., 2020), although their therapeutic activity in the neural context appears to derive primarily from immunomodulatory and trophic effects on the pathobiology rather than integration and cell replacement (Freedman et al., 2010; Dulamea, 2015; Wu et al., 2020).

Most of these studies have employed autologous BM-MSCs or AD-MSCs (occasionally in the form of a stromal vascular fraction, AD-SVF, a heterogeneous cell fraction including MSCs, HSCs, and various myeloid and lymphoid cells, amongst others), but allogeneic MSCs from umbilical cord or placental sources have also been trialed. A number of phase 1 clinical studies have demonstrated the feasibility and safety of the IV (Cohen et al., 2018; Feng et al., 2019; Iacobaeus et al., 2019) or IT (Mohyeddin Bonab et al., 2007; Sahraian et al., 2019) administration of BM-MSCs, although in one study a large combined IT and intracisternal dose of MSCs (100 × 10^6^ cells) was associated with transient encephalopathy, with seizures in one recipient (Yamout et al., 2010). In several of these pilot studies hints of therapeutic benefit were observed, but small enrolment sizes and a lack of controls preclude an assessment of efficacy.

An ongoing phase 1 study (NCT03069170) is comparing IV and IT BM-MSC administration routes with respect to primary outcomes of magnetic resonance imaging (MRI) metrics, safety, and functional changes, while a recent case report describes reduced radiological inflammatory activity and clinical stabilization in a RRMS patient receiving multiple IT and IV treatments over a period of 4 years (Hou et al., 2013). Notably, the patient received treatments of both autologous BM-MSCs and allogeneic UC-MSCs with adverse events reported only with the allogeneic treatment (Hou et al., 2013), but these were minor and may relate to the larger dosages employed in the IV UC-MSC transplants.

In other phase 1 clinical study of allogeneic sources serious but transient adverse events (including an anaphylactoid reaction and superficial thrombophlebitis) were reported in the highest-dose (600 × 10^6^ cells) IV placental MSC treatment group of a placebo-controlled study (Lublin et al., 2014), whereas only minor transient reactions (dizziness, headache, irritation) were reported in a very small study of multiple UC-MSC treatments (seven IV administrations per patient, each 1–6 × 10^6^ cells/kg body weight) (Meng et al., 2018). Both studies included participants with SPMS and generated some early evidence of disease stabilization and mitigation of clinical symptoms.

In phase 1 safety studies of autologous AD-SVFs, three IT administrations of up to 14.2 × 10^6^ cells did not lead to serious adverse events (Siennicka et al., 2016), whereas a study of participants receiving between 1 and 15 ICV cell administrations revealed several instances of treatment-related transient meningismus and additional complications related to the use of implanted conduits in delivering the cells (Duma et al., 2019). The safety of autologous endometrial MSCs administered IV to women with SPMS is also currently being explored in a placebo-controlled phase 1 study in Iran (Iranian Registry of Clinical Trials: IRCT20190711044175N1).

Studies of the efficacy of MSCs on MS have likewise involved both autologous and allogeneic sources, with a mix of IV and IT administration. Uncontrolled phase 1/2 studies have further affirmed the safety of the intervention, but with clinical outcomes ranging from no significant effect (Dahbour et al., 2017) through to short-to-intermediate-term stabilization of disease activity and/or progression (Stepien et al., 2016), modest improvements in function or quality of life (Riordan et al., 2018), and long-term reductions in relapse occurrence (Lu et al., 2020).

In a small, placebo-controlled phase 2 crossover clinical study of IV-delivered autologous BM-MSCs, evidence of non-significant decreases in MRI lesion activity and circulating Th1 cell counts is reported (Llufriu et al., 2014).

A further phase 2 study of multiple IV administrations of UC-MSCs found treatment-associated reductions in clinical scores and symptoms, and number of relapses, with serum analyses suggesting a shift from Th1-like (pro-inflammatory) to Th2-like (anti-inflammatory) immune responses in treated patients (Li et al., 2014).

The ambitious MEsenchymal StEm cells for Multiple Sclerosis (MESEMS) study, a placebo-controlled, crossover phase 1/2 study of autologous BM-MSCs, incorporated multiple partially independent studies from different centers under harmonized protocols to overcome funding constraints and improve statistical power (Uccelli et al., 2019). Preliminary results support the safety of the approach (single IV injection of 1–2 × 10^6^ MSCs per kg of body weight), but with no impact on the number of contrast-enhancing lesions by MRI at 24 weeks, the primary efficacy endpoint (Uccelli et al., 2020).

Any benefit of MSC transplantation in treating PMS specifically is often obfuscated by trial cohorts including both RRMS and PMS patients. Even those trials focused specifically on PMS often enroll participants with *active* PMS, a population apparently more responsive to the immunomodulatory effects of MSCs.

In a placebo-controlled phase 2 crossover study comparing the IT and IV administration of autologous BM-MSCs to participants with PPMS and SPMS, those having had a relapse or MRI activity in the year prior to treatment were more responsive to treatment (Petrou et al., 2020). Overall, the inhibition of disease progression and beneficial functional outcomes were observed, with IT administration eliciting a more significant effect than IV, and two MSC injections (at a 6-month interval) proving more efficacious than a single dose. This study was a follow-up to an earlier single-arm phase 1/2 trial of IT administration of autologous BM-MSCs to MS and amyotrophic lateral sclerosis patients in which acute immunomodulatory effects and a statistically significant improvement in EDSS scores were observed in the MS cohort during 6 months of follow-up (Karussis et al., 2010).

Other PMS-specific trials include a phase 2 study of IT autologous BM-MSC administration in which no significant improvements where observed in PMS patients (Bonab et al., 2012), and a placebo-controlled, dose-ranging phase 1/2 study of IV autologous AD-MSCs in which inconclusive signs of efficacy in SPMS were inferred from changes in the number of MRI lesions and evoked potential parameters (Fernandez et al., 2018).

On the other hand, a phase 1/2 study of combined IT and IV UC-MSCs in SPMS patients showed decreased relapse frequency and/or lesion severity, improvement in clinical scores, and evidence of peripheral immunomodulation (Lu et al., 2013). Similarly, a single-arm phase 1/2a study of IV autologous BM-MSCs in SPMS patients revealed improvements in visual acuity, reduction of visual evoked response latencies, and protection of the optic nerve area by optical coherence tomography (OCT) in the 6 months following transplantation (Connick et al., 2011, 2012).

Several additional randomized phase 1/2 trials have been completed but the results not yet reported, including placebo-controlled studies of IV-administered autologous BM-MSCs in RRMS patients and a crossover study in RRMS and SPMS participants, and a study comparing the beneficial effects of combined IT and IV administration of UC-MSCs (and a follow-up booster of MSC-conditioned media) with supervised physical therapy (Alghwiri et al., 2020).

Ongoing clinical studies include a unique phase 1 study (NCT02795052) comparing IV administration of autologous BM-MSCs with a combination of IV and intranasal delivery, currently recruiting participants across various neurological conditions, including MS (Weiss and Levy, 2016), as well as a larger, controlled phase 2/3 study of autologous BM-MSCs on RRMS relapse rates and clinical/radiological outcomes employing an initial IV dose followed up with an IT booster (IRCT20191004044975N1).

Progressive MS-specific trials currently underway include a phase 1/2 placebo-controlled crossover study of IT autologous BM-MSCs exploring neurophysiological, functional, and quality-of-life outcomes in both PPMS and SPMS patients (NCT04749667), a single-arm phase 1/2 studies of the effects autologous AD-MSCs clinical, radiological, and immunological measures in SPMS patients (NCT03696485 and IRCT20091127002778N1), and a single-arm phase 2 study of BrainStorm Cell Therapeutics’ NurOwn product (neurotrophic factor-expressing autologous BM-MSCs) upon multiple IT administration to PMS patients (NCT03799718).

In order to increase the regenerative potential of MSCs, a number of clinical studies by the Tisch MS Research Center of New York have employed MSC neural progenitors (MSC-NPs), a subpopulation of mesenchymal cells that exhibit neuroectodermal lineage characteristics and are likely to be more CNS compatible while retaining the immunomodulatory and trophic capabilities of common MSCs (Harris et al., 2012). The tolerability of the treatment was established by a phase 1 dose-escalating IT administration study in people with PMS, which supported feasibility and long-term safety with post-treatment improvement of clinical scores in 4 (out of 6) participants (Harris et al., 2016). A follow-up single-arm phase 1 study, comprising 3 IT treatments of MSC-NPs administered at 3-month intervals, found an improved mean EDSS score over 12 months of follow-up sustained by 7 (out of 20) participants at 2 years (Harris et al., 2018, 2021). Effects were found to be more pronounced in ambulatory (low/medium-disability) SPMS participants, rather than (non-ambulatory), PPMS participants. Clinical outcomes also included signs of improved muscle strength (70% of participants) and bladder function (50% of participants) (Harris et al., 2018), while cerebrospinal fluid biomarker changes, including a decrease in CCL2 and increases in IL-8, HGF, and CXCL12, were found to reflect treatment-related immunoregulatory and trophic effects (Harris et al., 2021). These outcomes have inspired an ongoing placebo-controlled phase 2 crossover trial (NCT03355365), which is recruiting 50 PMS participants to receive a total of 6 IT treatments at 2-month intervals, with functional outcomes (including clinical scores and bladder function) assessed over 36 months of follow-up. Participants in this trial are to be ambulatory (i.e., EDSS ≤ 6.5) and will be stratified according to baseline EDSS score and disease subtype (PPMS or SPMS); an expanded-access study for those that do not meet eligibility criteria is also planned (NCT03822858).

### Clinical Evidence for NSCs and Pluripotent Stem Cells in PMS

Two dose-response phase 1 safety and tolerability clinical studies of bona fide allogeneic fetal-derived NSC transplantation in PMS patients have been conducted.

Clinical trial NCT03269071 (IRCCS Ospedale San Raffaele) has enrolled 4 cohorts of 3 PMS participants each to receive an IT dose of 0.7–5.7 × 10^6^ cells/kg, with quality-of-life outcomes to be assessed, while trial NCT03282760 (Casa Sollievo della Sofferenza IRCCS) has treated 24 SPMS participants with an ICV dose of 5–24 × 10^6^ cells each, examining functional, cognitive, and neurophysiological changes post-treatment. Both trials have been completed but outcomes are yet to be reported at the time of writing.

While iPSC-derived cells have begun to see study in other clinical contexts [e.g., iPSC-derived NSCs in spinal cord injury (Nagoshi et al., 2020) and iPSC-derived retinal pigment epithelial cells for treating age-related macular degeneration (Mandai et al., 2017)], the technology has yet to undergo trials in MS despite its potential (Fossati and Douvaras, 2014). Concerns regarding the safety of pluripotent cells sources is likely to be a key factor impeding their clinical development (Ortuño-Costela et al., 2019), although case reports of *embryonic* stem cell transplants in MS patients have been published (Shroff, 2016). The use of iNSCs, generated by direct reprogramming of somatic cells and thus bypassing the pluripotent state, may represent an attractive alternative approach (Xie et al., 2016).

Thus, evidence to date supports the safety and feasibility of HSC and MSC therapies in MS, with immunomodulatory and/or trophic mechanisms of action providing modest, transient clinical benefits in RRMS or *active* PMS. More robust clinical studies and systematic reviews (see e.g., Rahim et al., 2019) will be required to establish the extent of these benefits and whether cell therapies provide advantages over current best standards of care. There is little current clinical evidence supporting the efficacy of HSC or MSC transplants in treating PMS, which will likely require novel mechanisms through which to not only halt disease progression but also foster CNS repair. NSCs, somatic or induced, may be key to providing the regenerative potential necessary to combat the gradual accumulation of disability arising from PMS, but clinical studies in this area are in their infancy.

## Conclusion

While the treatment of MS continues to advance, therapeutic options remain restricted to ameliorating or preventing relapses and acute inflammation events in RRMS or *active* PMS. There are no proven interventions able to halt the gradual accumulation of disability associated with PMS, let alone effectively promote repair of the damaged CNS. The prospect of stem cell therapy brings with it great promises of regenerative potential, yet the weight of preclinical and clinical evidence to date points to immunomodulatory and trophic effects that are most advantageous to addressing *relapsing/active* forms of the disease. The best hopes for an impact on PMS perhaps lay with NSCs, either somatic or transdifferentiated, which have given rise to compelling preclinical evidence for the amelioration of chronic neuroinflammation through novel mechanisms of action, but have yet to see substantial clinical study in the context of MS. Ultimately, while the safety and feasibility of stem cell transplantation has been demonstrated across various cell types and administration routes, there remains a need for larger and/or more rigorous studies to quantify the benefits of stem cell therapy and demonstrate an advantage over current best standards of care. Whether stem cell therapies have the potential to repair the PMS CNS in a clinical setting remains to be seen.

## Author Contributions

All authors contributed equally to the research, writing, and editing of this review.

## Conflict of Interest

SP is co-founder, CSO and shareholder (>5%) of CITC Ltd. and iSTEM Therapeutics, and co-founder and Non-Executive Director at Asitia Therapeutics. LP-J is shareholder of CITC Ltd. JAS is an employee of CITC Ltd. and Head of Research at iSTEM Tx. The remaining authors declare that the research was conducted in the absence of any commercial or financial relationships that could be construed as a potential conflict of interest.
