# Supplementary material for: Stem Cell Therapies for Progressive Multiple Sclerosis
Source: Front Cell Dev Biol. 2021 Jul 9;9:696434. doi: 10.3389/fcell.2021.696434 (PMC8299560; doi:10.3389/fcell.2021.696434)
Supplement: Supplementary file 1 [file Table_1.docx]

Supplementary Table 1. Clinical trials of non-hematopoietic stem cells in MS.

| **Cell Type** | **Sponsor/**  **Coordinator** | **Condition** | **Administration Route**  **(Cell Dose)** | **Study Design** | **Enrollment** | **Start** | **End^i^** | **Status^ii^ & Outcomes** | **Registration^iii^ and References** |
| --- | --- | --- | --- | --- | --- | --- | --- | --- | --- |
| Fetal human NSCs | IRCCS Ospedale San Raffaele (Italy) | PMS | IT  (0.7-5.4×10^6^ cells/kg body weight) | Phase 1  Open-label, sequential assignment, dose-ranging | 4 | 2017 | 2020 | Current status unknown  *Primary outcome measures*: feasibility, safety and tolerability up to 96 weeks; quality-of-life | NCT03269071 |
| Fetal human NSCs | Casa Sollievo della Sofferenza IRCCS (Italy) | SPMS | ICV  (5-24×10^6^ cells/participant) | Phase 1  Open-label, sequential assignment, dose-ranging, multicenter | 24 | 2017 | 2021 | Current status unknown  *Primary outcome measures*: feasibility, safety and tolerability up to 1 year.  *Secondary outcome measures*: functional, cognitive and neurophysiological changes, biomarkers, relapse rate. | NCT03282760 |
| Autologous BM-MSCs | Sina MS Research Center (Iran) | PPMS, SPMS | IT  (Mean dose of 8.7×10^6^ cells/participant) | Phase 1  Open-label, single-arm | 10  (8 SPMS, 2 PPMS) | -- | -- | Completed  No treatment-related SAEs. Modest improvements in EDSS scores and neurological function in some participants over mean follow-up period of 19 months. | N/A  (Mohyeddin Bonab et al., 2007) |
| Autologous BM-MSCs | American University of Beirut Medical Center (Lebanon) | SPMS | IT and IC  (32-52×10^6^ cells/participant; a single participant received 100×10^6^ cells) | Phase 1  Open-label, single-arm | 7 | -- | -- | Completed  Transient encephalopathy with seizures in patient receiving highest dose, otherwise no treatment-related SAEs. Trend of EDSS and visual function improvement over 6 months, but not radiological efficacy. | N/A  (Yamout et al., 2010) |
| Autologous BM-MSCs | MS Research Center, Neuroscience Institute, Tehran University of Medical Sciences (Iran) | RRMS, SPMS | IT  (Mean dose of 57×10^6^ cells/participant; 2 participants received a booster dose after 1 year) | Phase 1  Open-label, single-arm | 4  (1 RRMS, 3 SPMS) | -- | -- | Completed  No treatment-related SAEs. Stabilization of SPMS progression, no new/enhanced plaques by MRI. | N/A  (Sahraian et al., 2019) |
| Allogeneic UC-MSCs | Chinese Academy of Medical Sciences and Peking Union Medical College (China) | RRMS, SPMS | IV  (7 treatments of 1-6×10^6^ cells/kg body weight at 3 month intervals) | Phase 1  Non-treated control, dual-arm | 3  (1 RRMS, 2 SPMS) | -- | -- | Completed  No treatment-related SAEs. Some mitigation of clinical signs and symptoms, reduced MRI loci during 8 years of follow-up. Evidence of immunomodulation from gene expression changes in peripheral blood. | N/A  (Meng et al., 2018) |
| Autologous AD-SVF | Maria Sklodowska-Curie Memorial Cancer Center and Institute of Oncology (Poland) | RRMS, SPMS | IT  (3 treatments of mean 13.5×10^6^ cells/participant, administered at 3-month intervals) | Phase 1 | 20  (15 RRMS, 5 SPMS) | -- | -- | Completed  No treatment-related SAEs. | N/A  (Siennicka et al., 2016) |
| Autologous BM-MSC-derived Neural Progenitors | Tisch Multiple Sclerosis Research Center of New York (USA) | PPMS, SPMS | IT  (2-5 treatments of doses ranging from 5×10^3^ to 1.6×10^7^ cells, administered at intervals of 2 to 8 months) | Phase 1  Open-label, single-arm | 6  (2 PPMS, 4 SPMS) | 2005 | 2013 | Completed  No treatment-related SAEs. Feasibility and tolerability established. 4 of 6 participants show clinical improvement over mean follow-up of 7.4 years. | N/A  (Harris et al., 2016) |
| Autologous BM-MSCs | Hadassah Medical Organization (Israel) | MS | IT  (Mean dose 63.2×10^6^ cells/participant; 5 participants received an additional intravenous treatment, mean dose 24.5×10^6^ cells/participant) | Phase 1/2  Open-label, single-arm | 15 | 2006 | 2009 | Completed  No treatment-related SAEs. Acute immunomodulatory effect, statistically significant improvement in mean EDSS over 6 months. | NCT00781872  (Karussis et al., 2010) |
| Autologous BM-MSCs + Allogeneic UC-MSCs | Yan’an Hospital of Kunming Medical University (China) | RRMS | IV and IT  (10 treatments over four years, ranging from 1.5×10^5^ to 3.3×10^8^ cells each) | Case Report | 1 | 2006 | 2012 | Completed  No treatment-related SAEs. Minor adverse events experienced upon allogeneic transplant. Significant reduction in inflammatory activity associated with MSC treatment, with no further relapses or lesions over 4 years of treatment/follow-up. | N/A  (Hou et al., 2013) |
| Autologous BM-MSCs | University of Cambridge (UK) | SPMS | IV  (Mean dose 1.6×10^6^ cells/kg body weight) | Phase 1/2a  Open-label, single-arm | 10 | 2008 | 2010 | Completed  No treatment-related SAEs. Improvements in visual acuity, visual evoked response latency, and optic nerve area 6 months post-transplant. | NCT00395200  (Connick et al., 2011; Connick et al., 2012) |
| Autologous BM-MSCs | Sina MS Research Center (Iran) | PPMS, SPMS | IT  (Mean dose 29.5×10^6^ cells/participant) | Phase 2  Open-label, single-arm | 25  (2 PPMS, 23 SPMS) | 2008 | 2011 | Completed  No treatment-related SAEs. No substantial improvement in clinical course (EDSS) or MRI during 12-month follow-up. | N/A  (Bonab et al., 2012) |
| Allogeneic UC-MSCs | Drum Tower Hospital of Nanjing University Medical School (China) | RRMS, NMO | IV and IT  (2×10^7^ cells IV and 2×10^7^ cells IT, then an additional 2×10^7^ cells IV weekly for 3 weeks) | Phase 1/2  Open-label, single-arm | 10  (5 RRMS, 5 NMO) | 2009 | 2020 | Completed  Long-term (10-year) safety and feasibility evidence. Reduced annual relapse occurrence. | N/A  (Lu et al., 2020) |
| Allogeneic Placental-MSCs | Celgene Cellular Therapeutics (USA) | RRMS, SPMS | IV  (Low dose: 150×10^6^ cells/participant; high dose: 600×10^6^ cells/participant) | Phase 1b  Placebo-controlled, double-masked, triple-arm, dose-ranging, multicentre | 16  (10 RRMS, 6 SPMS) | 2010 | 2011 | Completed  Instances of anaphylactoid reaction and superficial thrombophlebitis with high-dose treatment, otherwise no treatment-related SAEs. All but one participant had stable or decreasing EDSS scores in the 6-12 months post-treatment. | (Lublin et al., 2014) |
| Allogeneic UC-MSCs | 148th Hospital (China) | RRMS, SPMS | IV  (3 treatments of 4×10^6^ cells/kg at two-week intervals) | Phase 2  Controlled, randomized, dual-arm | 23  (16 RRMS, 7 SPMS) | 2010 | 2012 | Completed  No treatment-related SAEs. Over 1-year follow-up: reduction in EDSS scores and number of relapses versus control group. Improvement in symptoms. Data supporting shift from Th1 to Th2 immunity. | N/A  (Li et al., 2014) |
| Autologous BM-MSCs | Albert Saiz, Hospital Clinic of Barcelona (Spain) | RRMS | IV  (1-2×10^6^ cells/kg body weight) | Phase 2  Placebo-controlled, double-masked, crossover (6 months) | 9 | 2010 | 2013 | Completed (recruitment terminated due to low enrollment accrual)  No treatment-related SAEs. Non-significant decrease in lesion measures by MRI at 6 and 12 months. Evidence supporting immunomodulatory effects. | NCT01228266  EudraCT: 2009-016442-74  (Llufriu et al., 2014) |
| Allogeneic UC-MSCs | Shenzhen Beike Bio-Technology Co., Ltd. (China) | SPMS | IV and IT  (2×10^7^ cells IV and 2×10^7^ cells IT, then an additional 2×10^7^ cells IV weekly for 3 weeks) | Phase 1/2  Open-label, single-arm | 8 | 2010 | 2012 | Completed  No treatment-related SAEs. Reduced relapse frequency and lesion severity during 18-month follow-up. Improvement in mean EDSS with evidence of peripheral immunomodulation. | NCT01364246  (Lu et al., 2013) |
| Autologous AD-MSCs | Andalusian Initiative for Advanced Therapies - Fundación Pública Andaluza Progreso y Salud (Spain) | SPMS | IV  (Low dose: 1×10^6^ cells/kg body weight; high dose: 4×10^6^ cells/kg body weight) | Phase 1/2  Placebo-controlled, multi-arm, quadruple-masked, multicenter | 30 | 2010 | 2015 | Completed  No treatment-related SAEs. Inconclusive evidence of efficacy by MRI and evoked potential measures in 12-month follow-up. | NCT01056471  EudraCT: 2008-004015-35  (Fernandez et al., 2018) |
| Autologous BM-MSCs | The Cleveland Clinic (USA) | RRMS, SPMS | IV  (Mean dose 1.9×10^6^ cells/kg body weight) | Phase 1  Open-label, single-arm | 24  (10 RRMS, 14 SPMS) | 2011 | 2014 | Completed  Treatment well-tolerated, no treatment-related SAEs in 6-month follow-up. | NCT00813969  (Cohen et al., 2018; Feng et al., 2019) |
| Autologous BM-MSCs | Royan Institute (Iran) | RRMS | IV  (*Dose unspecified*) | Phase 1/2  Placebo-controlled, double-masked | 22 | 2011 | 2014 | Completed  *Primary outcome measures*: MRI changes, relapse frequency, EDSS, and MSFC (6 months); brain atrophy (12 months).  *Secondary outcome measures*: QoL and safety (6 months). | NCT01377870 |
| Autologous BM-MSCs | Antonio Uccelli, University of Genova (Italy), other members of the international MESEMS^†^ study group (Uccelli et al., 2019) | RRMS, PPMS, SPMS | IV  (1-2×10^6^ cells/kg body weight) | Phase 1/2  Multicenter, placebo-controlled, quadruple-masked, crossover (week 24) | 144 | 2012 | 2020 | Completed  No treatment-related SAEs. No effect on primary outcome of contrast-enhancing lesions by MRI. | (Uccelli et al., 2020)  *Incorporates the following trials:*  NCT01854957 (EudraCT: 2011–001295-19)  EudraCT: 2012-000518-13  NCT01730547  NCT01606215 (EudraCT: 2012–002357-35)  NCT01745783  NCT02035514 (EudraCT: 2010–024081–21)  EudraCT: 2015-000137-78  NCT02239393 |
| Autologous BM-MSCs  (and conditioned media) | University of Jordan (Jordan) | RRMS, SPMS | IT  (Mean dose 11×10^7^ cells/participant; mean volume of 18 mL conditioned media administered 1 month post-transplant) | Phase 1/2  Open-label, single-arm | 10  (2 RRMS, 8 SPMS) | 2012 | 2016 | Completed  No treatment-related SAEs. No significant changes in clinical scores amongst most participants over 12-month follow-up; stable or worsening MRI and ophthalmological measures. | NCT01895439  (Dahbour et al., 2017) |
| Autologous BM-MSCs | Karolinska Institutet (Sweden) | PPMS, SPMS | IV  (1-2×10^6^ cells/kg body weight) | Phase 1  Open-label, single-arm | 7  (2 PPMS, 5 SPMS) | 2013 | 2016 | Completed  No treatment-related SAEs. Transient beneficial clinical effects (stable EDSS, lack of new T2 lesions) at 12 weeks post-transplantation, declining by 48 weeks. Early peripheral immunotolerogenic effects. | NCT03778333  (Iacobaeus et al., 2019) |
| Allogeneic UC-MSCs | Translational Biosciences (Panama) | RRMS, PPMS, SPMS | IV  (20×10^6^ cells/participant, daily for 7 days) | Phase 1/2  Open-label, single-arm | 20  (15 RRMS, 4 PPMS, 1 SPMS) | 2014 | 2016 | Completed  No treatment-related SAEs. Improved mean EDSS score over 12-month follow-up. Statistically-significant improvements in functional measures (bladder, bowel, sexual dysfunction, non-dominant hand, fatigue, and walk time). Improved QoL measures. Absence of disease progression or new/active lesions by MRI in most participants. | NCT02034188  (Riordan et al., 2018) |
| Autologous AD-MSCs | Military Institute of Medicine, Central Clinical Hospital of the Ministry of National Defense (Poland) | RRMS, SPMS | IT  (3 treatments of 12×10^6^ cells each, administered at 3-month intervals) | Phase 1/2  Open-label, single-arm | 20  (13 RRMS, 7 SPMS) | 2014 | 2016 | Completed  No treatment-related SAEs. No statistically significant improvements over 18 months of follow-up, but no disease progression or relapses. | N/A  (Stepien et al., 2016) |
| Autologous AD-SVF | Elliot Lander, Cell Surgical Network Inc. (USA) | PMS, other neurological conditions | ICV (via Ommaya reservoir or ventriculoperitoneal shunt)  (1-15 treatments of an average 1.6×10^6^ to 2.5×10^8^ cells each, administered every 2-3 months) | Phase 1  Open-label, single-arm | 6 PMS  (31 total) | 2014 | 2017 | Completed  Overall safety: several instances of transient meningismus following administration; one instance of hospitalization due to possible treatment-related acute hydrocephalus, three other hospitalizations within 30 days of treatment due to other factors.  EDSS outcomes (PMS participants): stabilization or improvement in all but 1 participant over a median follow-up time of 9.2 months. | N/A  (Duma et al., 2019) |
| Autologous BM-MSC-derived Neural Progenitors | Tisch Multiple Sclerosis Research Center of New York (USA) | PPMS, SPMS | IT  (3 treatments of up to 1×10^7^ cells each, administered at 3-month intervals) | Phase 1  Open-label, single-arm | 20  (4 PPMS, 16 SPMS) | 2014 | 2017 | Completed  No treatment-related SAEs. Improved median EDSS score over 12-month follow-up, with signs of improved muscle strength and bladder function. 7 participants showed sustained EDSS improvement at 2 year follow-up. CSF biomarker changes post-transplantation reflect immunoregulatory and trophic effects of treatment. | NCT01933802  (Harris et al., 2018; Harris et al., 2021) |
| Autologous BM-MSCs | Banc de Sang i Teixits (Spain) | RRMS, SPMS | IV  (*Dose unspecified*) | Phase 1/2  Placebo-controlled, quadruple-masked, crossover (6 months) | 8 | 2015 | 2018 | Completed  *Primary outcome measures*: Adverse events.  *Secondary outcome measures*: EDSS and lesion quantification by MRI over 12 months. | NCT02495766  EudraCT: 2012-000734-19 |
| Autologous BM-MSCs | Dimitrios Karussis, Hadassah Medical Organization (Israel) | PPMS, SPMS | IT or IV  (1-2×10^6^ cells/kg body weight) | Phase 2  Placebo-controlled, double-masked, crossover (6 months) | 48  (7 PPMS, 41 SPMS) | 2015 | 2018 | Completed  No treatment-related SAEs. Significant inhibition of disease progression versus placebo (IT > IV) during 12-month follow-up. IT transplant associated with positive functional, cognitive, and MRI outcomes. | NCT02166021  (Petrou et al., 2020) |
| Autologous AD-MSCs | Mashhad University of Medical Sciences (Iran) | SPMS | *Route unspecified*  (2 treatments of 2.5×10^6^ cells/kg body weight, administered 7 days apart) | Phase 1/2  Open-label, single-arm | 10 | 2016 |  | Recruitment complete  *Primary outcome measures*: safety (9 months).  *Secondary outcome measures*: clinical scores, MRI, peripheral Treg cell characterization/quantification (9 months). | IRCT20091127002778N1 |
| Autologous BM-MSCs | Stem Cells Arabia (Jordan) | RRMS | IV and IT  (*Dose unspecified*) | Phase 1  Open-label, single-arm | 50 | 2016 | 2021 | Recruiting  *Primary outcome measures*: MRI assessment (6 months); safety and EDSS assessments (12 months).  *Secondary outcome measures*: QoL and axonal effects by OCT (6 months); immunologic effects (1 month). | NCT03069170 |
| Autologous BM-MSCs | MD Stem Cells (USA) | Various Neurologic Disorders and Injuries | IV or IV and IN  (*Dose unspecified*) | Phase 1  Open-label, parallel-arm | 300 total | 2016 | 2023 | Recruiting  *Primary outcome measures*: Activities of daily living (3-12 months).  *Secondary outcome measures*: neurologic function (3-12 months). | NCT02795052  (Weiss and Levy, 2016) |
| Allogeneic UC-MSCs  (and conditioned media) | University of Jordan (Jordan) | MS | IT + IV  (2 treatments of 100×10^6^ cells IT + 50×10^6^ cells IV, 1 month apart; 8-10 mL conditioned media at month 3) | Phase 1/2  Active comparator-controlled (supervised physical therapy), single-masked, multi-arm (physical therapy, UC-MSCs, or both) | 60 | 2017 | 2020 | Completed  *Primary outcome measures*: Feasibility.  *Secondary outcome measures*: safety; functional outcomes, non-motor efficacy, biological measures (3-12 months). | NCT03326505  (Alghwiri et al., 2020) |
| Autologous BM-MSC-derived Neural Progenitors | Tisch Multiple Sclerosis Research Center of New York (USA) | PMS | IT  (6 treatments, dose unspecified, 2-month intervals) | Phase 2  Placebo-controlled, quadruple-masked, crossover (12 months) | 50 | 2018 | 2023 | Active, not recruiting  *Primary outcome measures*: EDSS score (36 months).  *Secondary outcome measures*: MSFC score and bladder function (36 months). | NCT03355365  (Expanded access: NCT03822858) |
| Autologous AD-MSCs | Stem Cell Medicine Ltd. (Israel) | SPMS | IT  (*Dose unspecified*) | Phase 1/2  Open-label, single-arm | 12 | 2019 | 2021 | Current status unknown  *Primary outcome measures*: adverse events (48 weeks).  *Secondary outcome measures*: lesion changes by MRI, EDSS score (24 weeks). | NCT03696485 |
| Autologous BM-MSCs secreting neurotrophic factors (MSC-NTF, “NurOwn”) | BrainStorm Cell Therapeutics (USA) | PMS | IT  (3 treatments of an unspecified dose) | Phase 2  Open-label, single-arm, multicenter | 20 | 2019 | 2021 | Active, not recruiting  *Primary outcome measures*: adverse events (28 weeks).  *Secondary outcome measures*: functional outcomes (T25FW or 9-HPT) at 28 weeks, changes in NTF concentration in CSF. | NCT03799718 |
| Autologous Endometrial MSCs | Tehran University of Medical Sciences (Iran) | SPMS | IV  (5×10^6^ cells/kg body weight) | Phase 1  Placebo-controlled, dual-arm | 10 | 2019 | -- | Recruiting  *Primary outcome measures:* safety (9 months).  *Secondary outcome measures*: clinical scores, neurological function by MRI, and B cell markers in peripheral blood (9 months). | IRCT20190711044175N1 |
| Autologous BM-MSCs | Vice-Presidency for Science and Technology (Iran) | RRMS | IV + IT  (IV dose of 1-2×10^6^ cells/kg body weight, followed 2 weeks later by 0.5×10^6^ cells/kg body weight IT dose) | Phase 2/3  Controlled, randomized, investigator-masked, dual-arm | 120 | 2019 | -- | Recruiting  *Primary outcome measures:* safety and number of clinical relapses (12 months).  *Secondary outcome measures*: MRI, EDSS and visual evoked potential changes (12 months). Progression-free survival. | IRCT20191004044975N1 |
| Autologous BM-MSCs | Haukeland University Hospital (Norway) | PPMS, SPMS | IT  (*Dose unspecified*) | Phase 1/2  Placebo-controlled, triple-masked, crossover (6 months) | 18 | 2021 | 2025 | Not yet recruiting  *Primary outcome measures:* neurophysiological parameters (combined evoked potentials) at 6 months.  *Secondary outcome measures*: neurophysiological parameters and MRI (12 months), EDSS, QoL, functional measures (T25FW and 9-HPT), ophthalmological outcomes (visual function and OCT), and adverse events (18 months). | NCT04749667 |

^i^End date as specified in trial registration database; ^ii^Status as specified in trial registration database (trials without updated statuses but with published results are noted as “Completed”); ^iii^Registration number on clinicaltrials.gov (NCT), European Union Drug Regulating Authorities Clinical Trials Database (EudraCT), or Iranian Registry of Clinical Trials (IRCT).

^†^MEsenchymal StEm cells for Multiple Sclerosis (MESEMS)

Alghwiri, A.A., Jamali, F., Aldughmi, M., Khalil, H., Al-Sharman, A., Alhattab, D., et al. (2020). The effect of stem cell therapy and comprehensive physical therapy in motor and non-motor symptoms in patients with multiple sclerosis: A comparative study. *Medicine* 99(34)**,** e21646. doi: 10.1097/md.0000000000021646.

Bonab, M.M., Sahraian, M.A., Aghsaie, A., Karvigh, S.A., Hosseinian, S.M., Nikbin, B., et al. (2012). Autologous mesenchymal stem cell therapy in progressive multiple sclerosis: an open label study. *Curr. Stem Cell Res. Ther.* 7(6)**,** 407-414.

Cohen, J.A., Imrey, P.B., Planchon, S.M., Bermel, R.A., Fisher, E., Fox, R.J., et al. (2018). Pilot trial of intravenous autologous culture-expanded mesenchymal stem cell transplantation in multiple sclerosis. *Mult. Scler.* 24(4)**,** 501-511. doi: 10.1177/1352458517703802.

Connick, P., Kolappan, M., Crawley, C., Webber, D.J., Patani, R., Michell, A.W., et al. (2012). Autologous mesenchymal stem cells for the treatment of secondary progressive multiple sclerosis: an open-label phase 2a proof-of-concept study. *Lancet Neurol.* 11(2)**,** 150-156. doi: 10.1016/s1474-4422(11)70305-2.

Connick, P., Kolappan, M., Patani, R., Scott, M.A., Crawley, C., He, X.L., et al. (2011). The mesenchymal stem cells in multiple sclerosis (MSCIMS) trial protocol and baseline cohort characteristics: an open-label pre-test: post-test study with blinded outcome assessments. *Trials* 12**,** 62. doi: 10.1186/1745-6215-12-62.

Dahbour, S., Jamali, F., Alhattab, D., Al-Radaideh, A., Ababneh, O., Al-Ryalat, N., et al. (2017). Mesenchymal stem cells and conditioned media in the treatment of multiple sclerosis patients: Clinical, ophthalmological and radiological assessments of safety and efficacy. *CNS Neurosci. Ther.* 23(11)**,** 866-874. doi: 10.1111/cns.12759.

Duma, C., Kopyov, O., Kopyov, A., Berman, M., Lander, E., Elam, M., et al. (2019). Human intracerebroventricular (ICV) injection of autologous, non-engineered, adipose-derived stromal vascular fraction (ADSVF) for neurodegenerative disorders: results of a 3-year phase 1 study of 113 injections in 31 patients. *Mol. Biol. Rep.* 46(5)**,** 5257-5272. doi: 10.1007/s11033-019-04983-5.

Feng, J., Offerman, E., Lin, J., Fisher, E., Planchon, S.M., Sakaie, K., et al. (2019). Exploratory MRI measures after intravenous autologous culture-expanded mesenchymal stem cell transplantation in multiple sclerosis. *Mult. Scler. J. Exp. Transl. Clin.* 5(2)**,** 2055217319856035. doi: 10.1177/2055217319856035.

Fernandez, O., Izquierdo, G., Fernandez, V., Leyva, L., Reyes, V., Guerrero, M., et al. (2018). Adipose-derived mesenchymal stem cells (AdMSC) for the treatment of secondary-progressive multiple sclerosis: A triple blinded, placebo controlled, randomized phase I/II safety and feasibility study. *PLoS ONE* 13(5)**,** e0195891. doi: 10.1371/journal.pone.0195891.

Harris, V.K., Stark, J., Vyshkina, T., Blackshear, L., Joo, G., Stefanova, V., et al. (2018). Phase I Trial of Intrathecal Mesenchymal Stem Cell-derived Neural Progenitors in Progressive Multiple Sclerosis. *EBioMedicine* 29**,** 23-30. doi: 10.1016/j.ebiom.2018.02.002.

Harris, V.K., Stark, J.W., Yang, S., Zanker, S., Tuddenham, J., and Sadiq, S.A. (2021). Mesenchymal stem cell-derived neural progenitors in progressive MS: Two-year follow-up of a phase I study. *Neurol. Neuroimmunol. Neuroinflamm.* 8(1)**,** e928. doi: 10.1212/nxi.0000000000000928.

Harris, V.K., Vyshkina, T., and Sadiq, S.A. (2016). Clinical safety of intrathecal administration of mesenchymal stromal cell-derived neural progenitors in multiple sclerosis. *Cytotherapy* 18(12)**,** 1476-1482. doi: 10.1016/j.jcyt.2016.08.007.

Hou, Z.L., Liu, Y., Mao, X.H., Wei, C.Y., Meng, M.Y., Liu, Y.H., et al. (2013). Transplantation of umbilical cord and bone marrow-derived mesenchymal stem cells in a patient with relapsing-remitting multiple sclerosis. *Cell Adh. Migr.* 7(5)**,** 404-407. doi: 10.4161/cam.26941.

Iacobaeus, E., Kadri, N., Lefsihane, K., Boberg, E., Gavin, C., Törnqvist Andrén, A., et al. (2019). Short and Long Term Clinical and Immunologic Follow up after Bone Marrow Mesenchymal Stromal Cell Therapy in Progressive Multiple Sclerosis-A Phase I Study. *J. Clin. Med.* 8(12). doi: 10.3390/jcm8122102.

Karussis, D., Karageorgiou, C., Vaknin-Dembinsky, A., Gowda-Kurkalli, B., Gomori, J.M., Kassis, I., et al. (2010). Safety and immunological effects of mesenchymal stem cell transplantation in patients with multiple sclerosis and amyotrophic lateral sclerosis. *Arch. Neurol.* 67(10)**,** 1187-1194. doi: 10.1001/archneurol.2010.248.

Li, J.F., Zhang, D.J., Geng, T., Chen, L., Huang, H., Yin, H.L., et al. (2014). The potential of human umbilical cord-derived mesenchymal stem cells as a novel cellular therapy for multiple sclerosis. *Cell Transplant.* 23 Suppl 1**,** S113-122. doi: 10.3727/096368914x685005.

Llufriu, S., Sepulveda, M., Blanco, Y., Marin, P., Moreno, B., Berenguer, J., et al. (2014). Randomized placebo-controlled phase II trial of autologous mesenchymal stem cells in multiple sclerosis. *PloS one* 9(12)**,** e113936. doi: 10.1371/journal.pone.0113936.

Lu, Z., Zhao, H., Xu, J., Zhang, Z., Zhang, X., Zhang, Y., et al. (2013). Human Umbilical Cord Mesenchymal Stem Cells in the Treatment of Secondary Progressive Multiple Sclerosis. *J. Stem Cell Res. Ther.* S6:002. doi: 10.4172/2157-7633.S6-002.

Lu, Z., Zhu, L., Liu, Z., Wu, J., Xu, Y., and Zhang, C.J. (2020). IV/IT hUC-MSCs Infusion in RRMS and NMO: A 10-Year Follow-Up Study. *Front. Neurol.* 11**,** 967. doi: 10.3389/fneur.2020.00967.

Lublin, F.D., Bowen, J.D., Huddlestone, J., Kremenchutzky, M., Carpenter, A., Corboy, J.R., et al. (2014). Human placenta-derived cells (PDA-001) for the treatment of adults with multiple sclerosis: a randomized, placebo-controlled, multiple-dose study. *Mult. Scler. Relat. Disord.* 3(6)**,** 696-704. doi: 10.1016/j.msard.2014.08.002.

Meng, M., Liu, Y., Wang, W., Wei, C., Liu, F., Du, Z., et al. (2018). Umbilical cord mesenchymal stem cell transplantation in the treatment of multiple sclerosis. *Am. J. Transl. Res.* 10(1)**,** 212-223.

Mohyeddin Bonab, M., Yazdanbakhsh, S., Lotfi, J., Alimoghaddom, K., Talebian, F., Hooshmand, F., et al. (2007). Does mesenchymal stem cell therapy help multiple sclerosis patients? Report of a pilot study. *Iran. J. Immunol.* 4(1)**,** 50-57.

Petrou, P., Kassis, I., Levin, N., Paul, F., Backner, Y., Benoliel, T., et al. (2020). Beneficial effects of autologous mesenchymal stem cell transplantation in active progressive multiple sclerosis. *Brain* 143(12)**,** 3574-3588. doi: 10.1093/brain/awaa333.

Riordan, N.H., Morales, I., Fernandez, G., Allen, N., Fearnot, N.E., Leckrone, M.E., et al. (2018). Clinical feasibility of umbilical cord tissue-derived mesenchymal stem cells in the treatment of multiple sclerosis. *J. Transl. Med.* 16(1)**,** 57. doi: 10.1186/s12967-018-1433-7.

Sahraian, M.A., Mohyeddin Bonab, M., Baghbanian, S.M., Owji, M., and Naser Moghadasi, A. (2019). Therapeutic Use of Intrathecal Mesenchymal Stem Cells in patients with Multiple Sclerosis: A Pilot Study with Booster Injection. *Immunol. Invest.* 48(2)**,** 160-168. doi: 10.1080/08820139.2018.1504301.

Siennicka, K., Zolocinska, A., Stepien, K., Lubina-Dabrowska, N., Maciagowska, M., Zolocinska, E., et al. (2016). Adipose-Derived Cells (Stromal Vascular Fraction) Transplanted for Orthopedical or Neurological Purposes: Are They Safe Enough? *Stem Cells Int.* 2016**,** 5762916. doi: 10.1155/2016/5762916.

Stepien, A., Dabrowska, N.L., Maciagowska, M., Macoch, R.P., Zolocinska, A., Mazur, S., et al. (2016). Clinical Application of Autologous Adipose Stem Cells in Patients with Multiple Sclerosis: Preliminary Results. *Mediators Inflamm.* 2016**,** 5302120. doi: 10.1155/2016/5302120.

Uccelli, A., Laroni, A., Brundin, L., Clanet, M., Fernández, Ó., Nabavi, S.M., et al. (2020). MEsenchymal StEm cells for Multiple Sclerosis (MESEMS) study: results from a multi-center, randomized, double blind, cross-over phase 2 clinical trial with autologous Mesenchymal Stem Cells (MSC) for the therapy of multiple sclerosis. *Eur. J. Neurol.* 27(Suppl. 1)**,** 27.

Uccelli, A., Laroni, A., Brundin, L., Clanet, M., Fernandez, O., Nabavi, S.M., et al. (2019). MEsenchymal StEm cells for Multiple Sclerosis (MESEMS): a randomized, double blind, cross-over phase I/II clinical trial with autologous mesenchymal stem cells for the therapy of multiple sclerosis. *Trials* 20(1)**,** 263. doi: 10.1186/s13063-019-3346-z.

Weiss, J., and Levy, S. (2016). Neurologic Stem Cell Treatment Study (NEST) using bone marrow derived stem cells for the treatment of neurological disorders and injuries: study protocol for a nonrandomized efficacy trial. *Clin. Trials Degen. Dis.* 1(4)**,** 176-180. doi: 10.4103/2468-5658.196984.

Yamout, B., Hourani, R., Salti, H., Barada, W., El-Hajj, T., Al-Kutoubi, A., et al. (2010). Bone marrow mesenchymal stem cell transplantation in patients with multiple sclerosis: a pilot study. *J. Neuroimmunol.* 227(1-2)**,** 185-189. doi: 10.1016/j.jneuroim.2010.07.013.
